# Assessment of renal function and prevalence of acute kidney injury following coronary artery bypass graft surgery and associated risk factors: A retrospective cohort study at a tertiary care hospital in Islamabad, Pakistan

**DOI:** 10.1097/MD.0000000000035482

**Published:** 2023-10-20

**Authors:** Muhammad Sajid Rafiq Abbasi, Khawar Sultan, Rukhsana Manzoor, Awais Ahmad Nizami, Naeem Ullah, Adnan Mushtaq, Humayun Saleem, Qudsia Umaira Khan, Amna Akbar, Sarosh Khan Jadoon, Sabahat Tasneem, Mohammad Saleem Khan, Sarosh Alvi

**Affiliations:** a FRCP Glasgow UK, Department of Nephrology, Pakistan Institute of Medical Sciences, Islamabad, Pakistan; b Rawal Institute of Health Sciences, Islamabad, Pakistan; c IMT-2, East Kent University Hospital, Canterbury, United Kingdom; d Director Cath Lab, Department of Cardiology, Shahida Islam Institute of Cardiology, Bahawalpur, Pakistan; e Post Graduate Resident Nephrology, Pakistan Institute of Medical Sciences, PIMS, Islamabad, Pakistan; f Registrar Nephrology, Pakistan institute of Medical Sciences (PIMS) Islamabad, Islamabad, Pakistan; g Health Services Academy, Islamabad, Pakistan; h CMH Lahore Medical College, Lahore, Pakistan; i District Headquarter Hospital Jhelum Valley, Muzaffarabad AJK, Pakistan; j Resident Surgeon SKBZ/CMH, Muzaffarabad AJK, Pakistan; k Public Health Professional, Health Services Academy, Islamabad, Pakistan; l DHQ Teaching, Hospital Kotli AJK, Kotli, Pakistan; m Teaching Faculty, University of Khartoum, Khartoum, Sudan.

**Keywords:** acute renal failure, cardiac surgery, retrospective cohort study, serum creatinine

## Abstract

Acute kidney injury (AKI) is a sudden decline in renal function after cardiac surgery. It is characterized by a significant reduction in glomerular filtration rate, alterations in serum creatinine (S.Cr) levels, and urine output. This study aimed to retrospectively analyze a cohort of 704 patients selected using stringent inclusion and exclusion criteria. AKI was defined by an increase of 0.3 mg/dL in S.Cr levels compared to baseline. Data were collected from the hospital and analyzed using SPSS 16.0. Data analysis revealed that 22% (n = 155) of the patients developed AKI on the second post-operative day, accompanied by a substantial increase in S.Cr levels (from 1.064 ± 0.2504 to 1.255 ± 0.2673, *P* < .000). Age and cardiopulmonary bypass duration were identified as risk factors along with ejection fraction and days of hospital stay, contributing to the development of AKI. Early renal replacement therapy can be planned when the diagnosis of AKI is established early after surgery.

## 1. Introduction

Coronary artery bypass graft (CABG) surgery is a common surgical procedure (0.4 million surgeries/annum)^[1]^ with good survival rate and reduced cardiovascular complications.^[2]^ Acute kidney injury (AKI) is defined as a sudden decline in renal function following cardiac surgery with an increase in serum creatinine (S.Cr), decrease in urine output, or both.^[3]^ It poses a long-term risk for cardiac failure, post-operative stroke, mediastinitis,^[4]^ damage to other organs,^[5]^ and a 2-fold risk of early mortality.^[6]^ Prevalence of AKI in the literature is up to 30%. Multiple mechanisms are involved in AKI pathophysiology. Kidney damage is caused by a combination of hemodynamic, inflammatory, and nephrotoxic factors.^[7]^ AKI must be identified as soon as possible to prevent progression.^[8]^ The time taken for S.Cr levels to rise following kidney injury may delay renal replacement therapy (RRT).^[9]^

A systematic evaluation of pre- and post-operative creatinine suggests that a 20% post-operative rise in plasma creatinine levels following cardiac surgery is a common finding. It significantly affects post-operative outcomes, particularly in the presence of multiorgan damage.^[10]^ It is useful to determine the severity of an AKI so that RRT can be considered at the earliest. Previously, clinical research has explored the risk factors associated with AKI. To utilize these observations and calculations for the effective implementation of interventions, we attempted to ascertain the risk factors associated with the occurrence of AKI in a Pakistani hospital in patients undergoing coronary artery bypass surgery.

## 2. Materials and methods

This retrospective cohort study investigated potential risk factors associated with the development of AKI after CABG surgery.

### 2.1. Ethical approval and consent to participate

The Cardiology Department at tertiary care hospitals in Islamabad, Pakistan, provided hospital records and data pertaining to patients who had undergone CABG surgery from January 2018 to December 2022. Direct communication was established with the administration of hospitals, and the research was conducted after prerequisite clearance from the Research and Ethics Committee of the involved institute (letter number: F-1-1/2018/ERB/SZABMU).

### 2.2. Inclusion criteria and exclusion criteria

**Figure 1.**
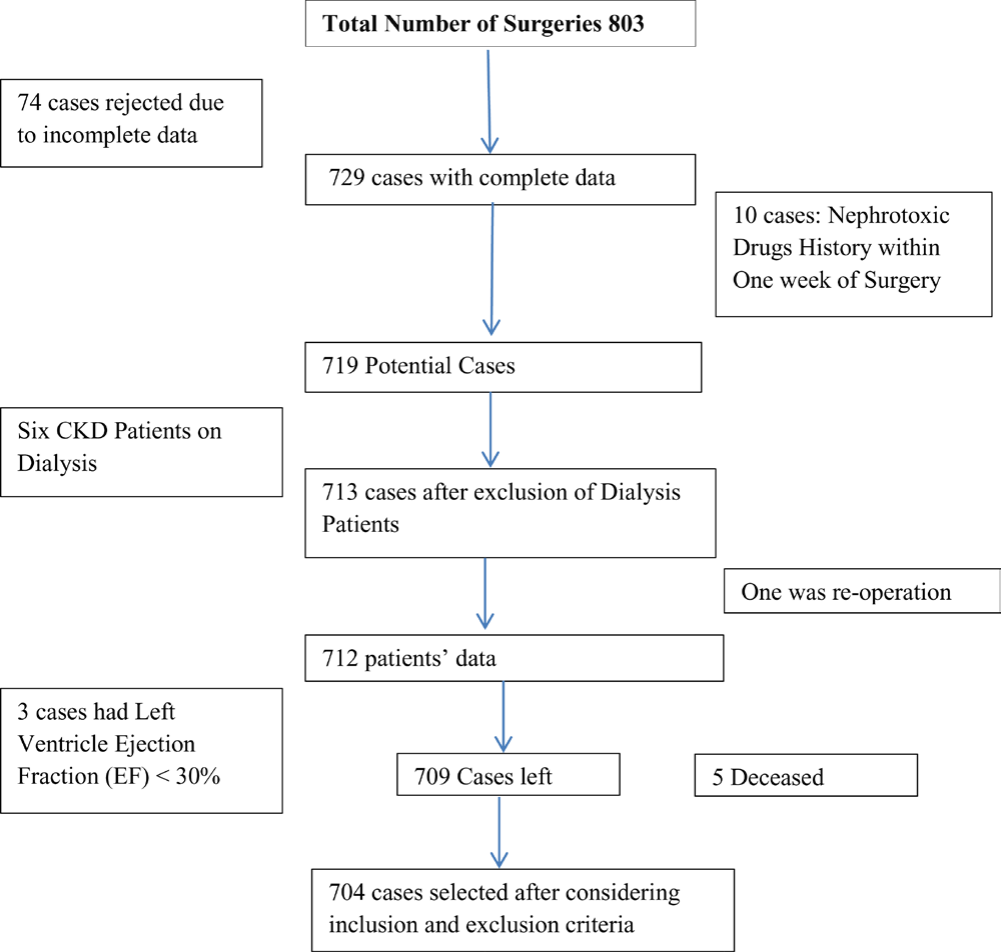
Inclusion and exclusion of cases.

The inclusion criteria were as follows: adult patients aged 18 years and older undergoing elective CABG surgery, pre-operative S.Cr levels of <2 mg/dL, a left ventricular ejection fraction (LVEF) of more than 30%, and availability of hospital records. The researchers excluded patients who died within 48 hours of surgery, individuals undergoing dialysis prior to surgery, patients who developed sepsis and those with a history of nephrotoxic medication use within a week before surgery. An elective CABG procedure was performed on 803 patients during the study period. Data from 704 patients were included in the present study after careful examination of each patient inclusion and exclusion criteria. A total of 74 cases were excluded from the analysis due to missing data, and an additional 10 patients data were disqualified as the patients had used nephrotoxic medications within 1 week before surgery. Six patients with chronic renal disease who were undergoing dialysis were excluded. One patient required reoperation, leading to exclusion from the dataset. Three individuals were excluded because their LVEF was <30%. Five patients died within 48 hours of surgery; one of them experienced recurrent ventricular tachycardia episodes and died following extubation despite cardiopulmonary resuscitation; the second patient suffered from stroke with unsuccessful cardiopulmonary resuscitation efforts for 37 minutes; the third patient succumbed to an ischemic stroke following extubation; and the fourth and fifth patients developed post-operative pneumonia and effusion (Fig. 1).

### 2.3. Clinical and socio-demographic factors

Demographic and physiological characteristics, clinical observations, pre-operative laboratory results, aortic cross-clamp (AC) time, and cardiopulmonary bypass (CPB) time were meticulously obtained from hospital records. The anesthesiologist operating room and intensive care unit records were carefully consulted to provide specific information on intubation, extubation, and CBP time for each patient. The hospital electronic database provides valuable information on previous disease histories and smoking status. Comprehensive details of prevalent conditions, such as diabetes mellitus (DM), hypertension (HTN), ischemic heart disease, cerebrovascular accident (CVA), chronic kidney disease (CKD), and asthma, were collected systematically. The researcher assigned appropriate labels to these variables based on globally recognized standards. For instance, patients who were using oral hypoglycemic medications or were insulin-dependent were categorized as having DM. Participants using medications for HTN prescribed by a physician were categorized as hypertensive. Mean and standard deviations of the continuous variables and categories on basis of median (Supplementary Table 1, http://links.lww.com/MD/K159) were determined in order to make analysis more comprehensive.

### 2.4. Classification and staging of acute kidney injury

A prediction model based on laboratory findings among patients undergoing cardiac surgery has good predictive accuracy for moderate-to-severe acute renal injury within 72 hours and 14 days after the procedure.^[9]^ S.Cr level is a sensitive indicator of AKI. The incidence of AKI was determined using the acute kidney injury network (AKIN) classification. AKI was diagnosed by a threshold value of S.Cr increasing by 0.3 mg/dL on the second post-surgical day. AKI staging was determined by considering both S.Cr and estimated glomerular filtration rate (eGFR) values. Stage 1 AKI is defined by AKIN standards as a 0.3 mL increase over baseline S.Cr levels.^[11]^ S.Cr levels were evaluated at the time closest to the surgical procedure, and these measurements were used as baseline values to determine the incidence of AKI. Subsequently, laboratory tests were conducted again on the second day after the surgical procedure, as well as on the seventh day.

Furthermore, laboratory investigations were performed on the follow-up day, wherein the values of both serum Cr and eGFR were assessed to confirm the persistence or resolution of AKI. Instead of depending solely on the risk, injury, failure, loss, end stage renal disease criteria (Supplementary Table 2, http://links.lww.com/MD/K160), the AKIN classification (Supplementary Table 3, http://links.lww.com/MD/K161) may facilitate the identification of patients who are at a higher risk of early death due to AKI. A rise in S.Cr of <0.3 mg/dL (26.5 mol/L) in <48 hours, a rise in S.Cr to <1.5 times baseline over the past 7 days, and a urine volume of 0.5 mL/kg/hour for 6 hours can be diagnosed as AKI.^[3,12–16]^ The kidney disease improving global outcomes classification can also be applied, but we did not use it in the present study.^[17]^ Using the AKI classification, the patients were then categorized into 2 distinct groups, with “1” indicating the presence of AKI and “0” representing the absence of AKI. A comparative analysis of the risk factors was conducted between the 2 groups. The extent of kidney damage was determined according to eGFR values, as S.Cr is not a good indicator of the severity of kidney damage. The spectrum of renal functional alterations extends from normal function, which is commonly indicated by an eGFR value of 90 mL/minute/1.73 m^2^ or higher, to the point of kidney failure, which is defined as an eGFR value below 15 mL/minute/1.73 m^2^ (Supplementary Table 4, http://links.lww.com/MD/K162). The eGFR values were computed from the S.Cr using an online historical calculator.^[18]^

### 2.5. Statistical analysis

Statistical analyses were performed using SPSS 16.0. For all continuous variables, age, BMI, median, mean, and standard deviation were employed. To simplify the analysis in SPSS, the median value was utilized as a cutoff point, and continuous data were categorized based on this cutoff value. Categorizing such data allows for an easier comparison of the characteristics of the study population. For example, age was divided into 2 categories: <60 and > 60 years. This division allows comparison of the characteristics between those under 60 years of age and those above 60 years of age, and allows statistical tests to identify significant factors associated with each category to be employed efficiently. After categorization, the frequencies and percentages of each variable were determined using frequency analysis (Supplementary Table 5, http://links.lww.com/MD/K163).

The chi-squared test was used for categorical variables. Statistical significance was set at *P* < .05, indicating an association between the tested variables. Variables with a *P* value of ≤ .2 in the comparative analysis (binary regression analysis) were selected for multinomial regression analysis. Multinomial regression analysis was conducted to investigate multiple predictors of AKI. A *P* value < .05 was considered significant and indicative of a positive association. Bias was mitigated by strictly adhering to inclusion and exclusion criteria. Kaplan-Meir curves were employed to validate the significance of the risk factors. The time variables used to generate the Kaplan–Meier curves were age (in years), hospital stay (in days), and follow-up (in weeks) (Supplementary Table 6, http://links.lww.com/MD/K164, Supplementary Table 7, http://links.lww.com/MD/K165).

Some of the calculations were performed using a Microsoft Excel worksheet, including the determination of percentages for the AKI and non-AKI groups, as well as the calculation of the percentage decline in eGFR values and the fractional increase in S.Cr values. Subsequently, the calculated percentages for the eGFR decrease and fractional increase in S.Cr were imported into SPSS for further analysis. These values were used to determine the stage of AKI according to the risk, injury, failure, loss, end stage renal disease criteria.

## 3. Results

Descriptive statistics: frequencies were calculated to analyze pre-operative categorical variables. Among the 704 participants, 84.4% (n = 594) were males, making this the predominant sex in this study population. Of all male participants, 127 (21.75%) developed AKI on the second day following surgery. Females constituted 15.6% (n = 110) of the total study population, with 25.5% (n = 28) experiencing AKI on the second postoperative day. Means and standard deviations were calculated for continuous variables to determine central tendency and data dispersion. The mean age was 59.74 ± 7.94 years, the average duration of hospital stay was 7.54 ± 3.09 days, and the mean BMI was 27.52 ± 3.79 kg/m^2^. The medians were calculated to determine central dispersion. Table 1

**Table 1:** Descriptive statistics and association of acute kidney injury (AKI) with demographics, preclinical and clinical characteristics of the Patients (*P* values).

Variable	Descriptive statistics			
	Mean ± SD			
^1^Age	59.74 ± 7.9			
^2^DIH	7.54 ± 3.09			
^3^BMI kg/m^2^	27.52 ± 3.8			
^4^EF % age	54.3 ± 13.04			
^5^AC time	38.19 ± 9.5			
^6^CPB time	57.77 ± 14.6			
Variable	Frequency (percentage)	Chi-square test (*P* values)		
		AKI at d 2	AKI at d 7	AKI at follow-up d
		155 (22%)	236 (33.5%)	394 (56%)
Gender				
Male	594 (84.4%)	.344	.394	.114
Female	110 (15.6%)			
Diagnosis				
TVCAD	471 (70%)	.474	.093	.175
TVCAD + LMS	233 (30%)			
Hypertension (HTN)				
Present	477 (67.8%)	.694	.225	.511
No	227 (32.2%)			
Diabetes mellitus (DM)				
Present	411 (58.5%)	.167	.773	.123
No	293 (41.5%)			
Ischemic heart disease (IHD)				
Present	503 (71.4%)	.725	.414	.800
No	201 (28.6%)			
Chronic kidney disease (CKD)				
Present	41 (6%)	.431	.800	.054
No	663 (94%)			
Asthma				
Present	10 (1.4%)	.356	.812	.368
No	694 (98.6%)			
Smoking				
Smoker	323 (46%)	.699	.398	.673
Nonsmoker	381 (54%)			
Cerebro vascular accident (CVA)				
Present	21 (3%)	.071	.652	.737
No	683 (97%)			
Age (yr)				
<60 yr	385 (54.7%)	.491	.146	.000
>60 yr	319 (45.3%)			
Body mass index (kg/m^2^)				
<27 kg/m^2^	340 (48.3%)	.349	.422	.241
>27 kg/m^2^	364 (51.7%)			
Aortic cross clamp (AC) Time (min)				
<37 min	362 (51.4%)	.392	.487	.715
>37 min	342 (48.6%)			
Cardiopulmonary bypass (CBP) Time (min)				
<58 min	375 (53.3%)	.231	.283	.032
>58 min	329 (46.7%)			
Left ventricular ejection fraction (% age)				
<55 %	397 (56.4%)	.079	.000	.666
>55%	307 (43.6%)			
Stay at hospital (d)				
<7 d	464 (66%)	.322	.035	.005
>7 d	240 (34%)			

○ Age in yr^1^.

○ Days in hospital (DIH)^2^.

○ Body mass index (BMI kg/m^2^)^3^.

○ Left ventricular ejection fraction (EF% age)^4^.

○ Aortic cross clamp (AC) Time (min)^5^.

○ Cardiopulmonary bypass (CPB) Time (min)^6^.

○ Prevalence of AKI was determined on basis of increase in Serum Creatinine values.

○ The *P* value for each variable was determined by performing a 2*2 contingency/chi-square test in SPSS, taking each variable against AKI prevalence one by one.

○ AKI_d 2: acute kidney injury (AKI) prevalence measured by an increase in serum creatinine values on d 2 following surgery

○ AKI_d 7: acute kidney injury (AKI) prevalence measured by increase in serum creatinine values on d 7 following surgery

○ AKI_Follow Up_d: acute kidney injury (AKI) prevalence measured by increase in serum creatinine values on follow-up d

○ Minimum and maximum value of variable in dataset, mean and standard deviation and median values of all the continuous variables are given Table 1, and categories of continuous variables in Table 2 in Supplementary material.

**Figure 2.**
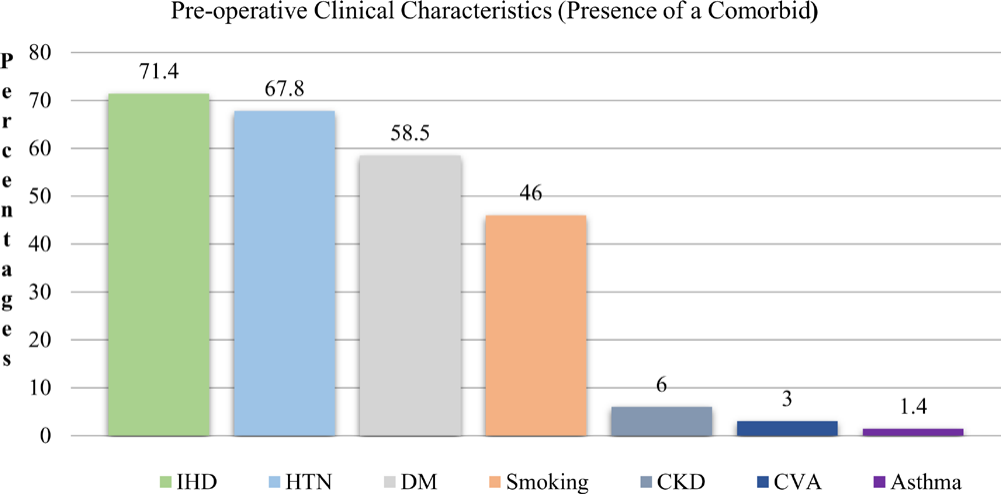
Pre-operative risk factors: IHD = ischemic heart disease, HTN = hypertension, DM = diabetes mellitus, CVA = cerebral vascular accident, CKD = chronic kidney disease.

Comorbidities: the comorbid and pre-operative risk factors investigated in this study included HTN, DM, ischemic heart disease, CKD, CVA, asthma, and smoking. The prevalence of DM was 58.5%, 67.8%, and 71.4%, respectively. 46 Of the participants, 46% were smokers and 51.7% had a BMI exceeding the mean value (Fig. 2).

Prevalence of AKI: AKI developed in 155 (22%) patients on post-operative day after surgery. The day 7 laboratory values showed that 236 (33.5%) patients developed AKI and 468 (66.5%) did not develop AKI (Table 1).

2*2 Contingency table: a chi-square test was conducted in SPSS to compare AKI-positive and AKI-negative groups with categorical variables. Days in the hospital were positively associated with AKI on day 7 after surgery (*P* < .035). Age, ejection fraction, and CPB time were also significantly associated (Table 1).

**Figure 3.**
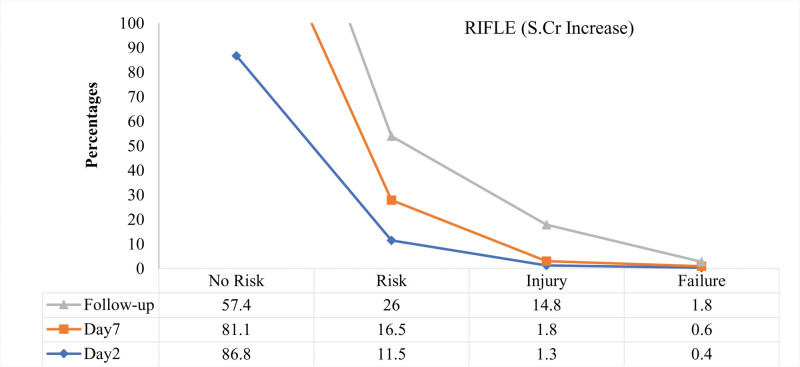
Acute kidney injury according to RIFLE (risk, injury, failure, loss, and end stage kidney disease) criteria on basis of fractional increase in S.Cr (serum creatinine) values measured on post-surgical d 2, d 7 and on follow-up d.

**Figure 4.**
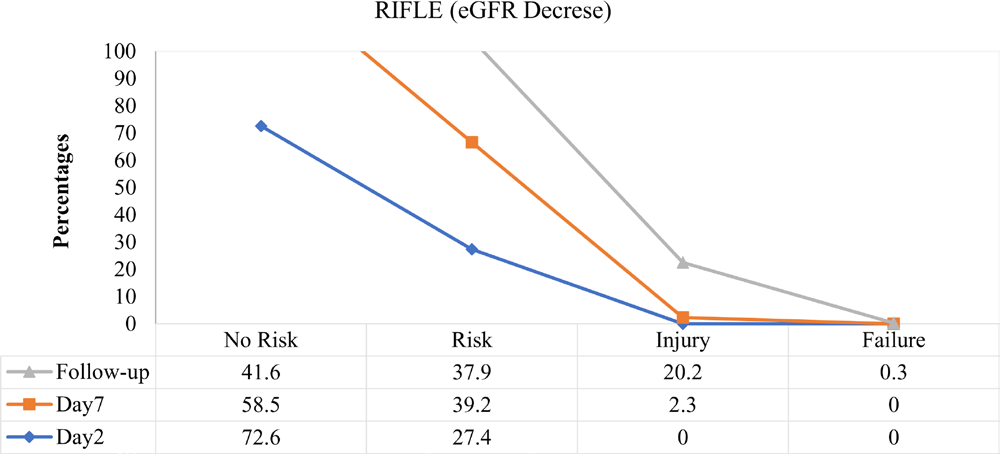
Acute kidney injury according to RIFLE (risk, injury, failure, loss and end stage kidney disease) criteria on basis of percentage decrease in estimated glomerular filtration rate (eGFR) values measured on post-surgical d 2, d 7 and on follow-up d.

Renal failure was defined as a 25%, 50%, or 75% decline in eGFR.^[19]^ The present analysis identified 27.4% (n = 193) of cases in stage 1 AKI (risk) on post-surgical day 2 as determined by eGFR, while it was 11.5% (n = 81) when determined by S.Cr values (Figs. 3 and 4), respectively.

**Figure 5.**
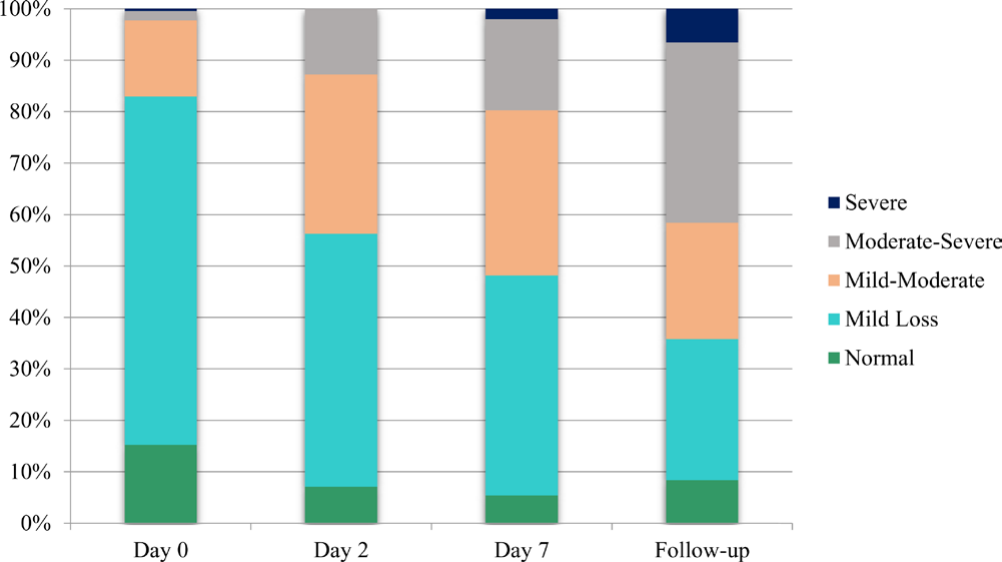
Chronic kidney disease staging according to estimated glomerular filtration rate (eGFR) categories (eGFR values measured on post-surgical d 2, d 7, and on follow-up d).

**Figure 6.**
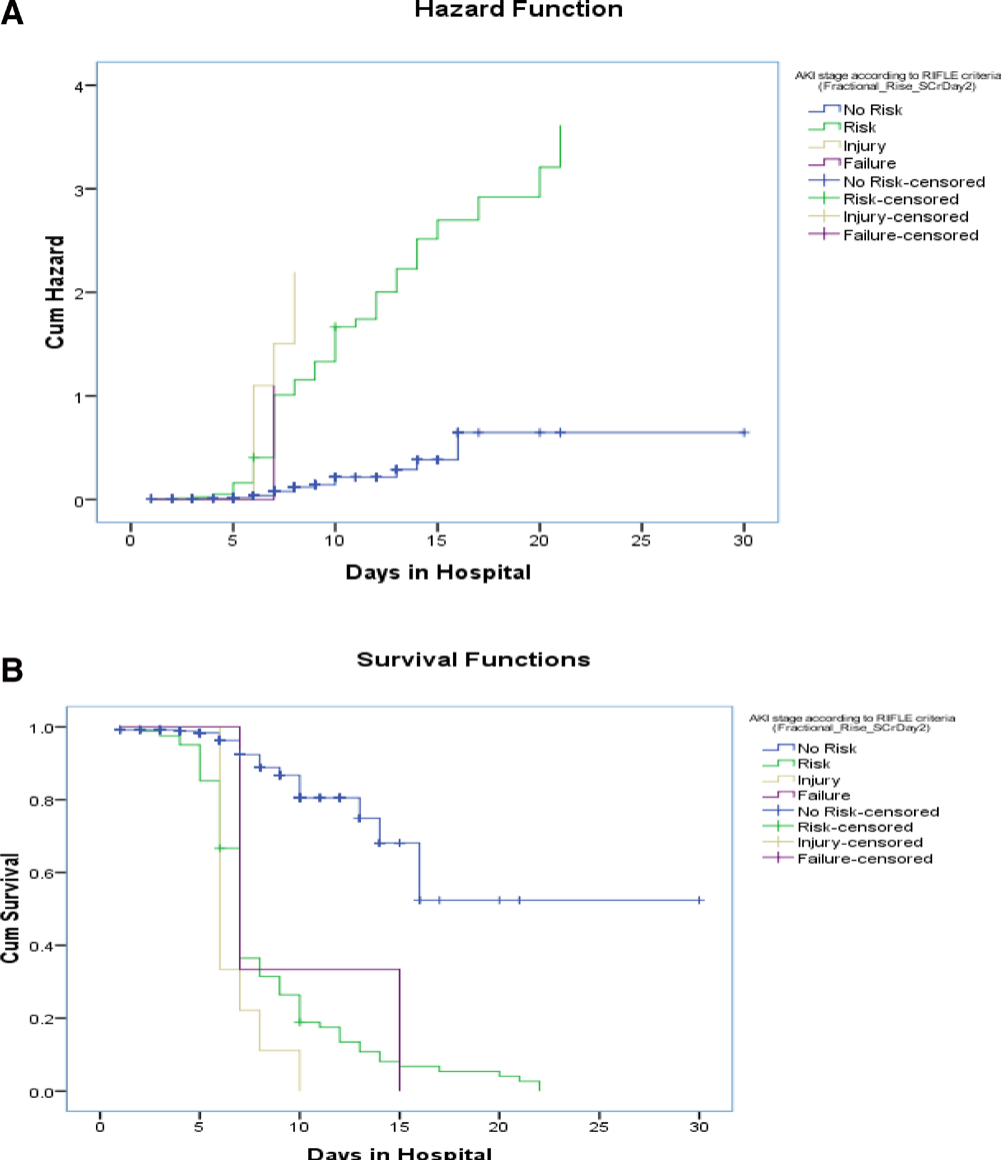
Test of equality of survival distributions (on d in hospital) for the different levels of acute kidney injury (AKI) stage according to risk, injury, failure, loss, end stage renal disease (RIFLE) criteria (Fractional_Rise_SCrDay2).

**Figure 7.**
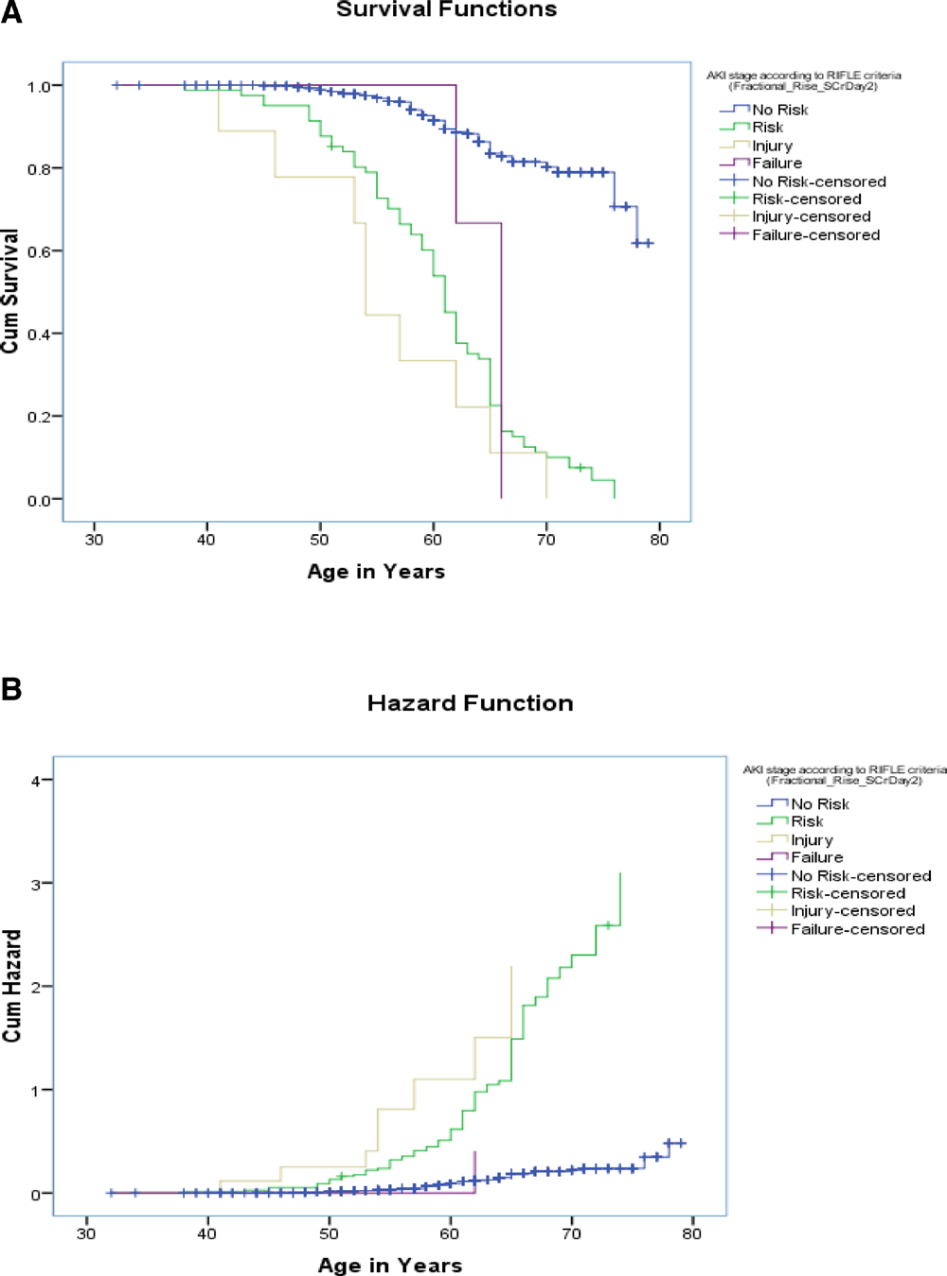
Test of equality of survival distributions (on age in yr) for the different levels of acute kidney injury (AKI) stage according to risk, injury, failure, loss, end stage renal disease (RIFLE) criteria (Fractional_Rise_SCrDay2).

**Figure 8.**
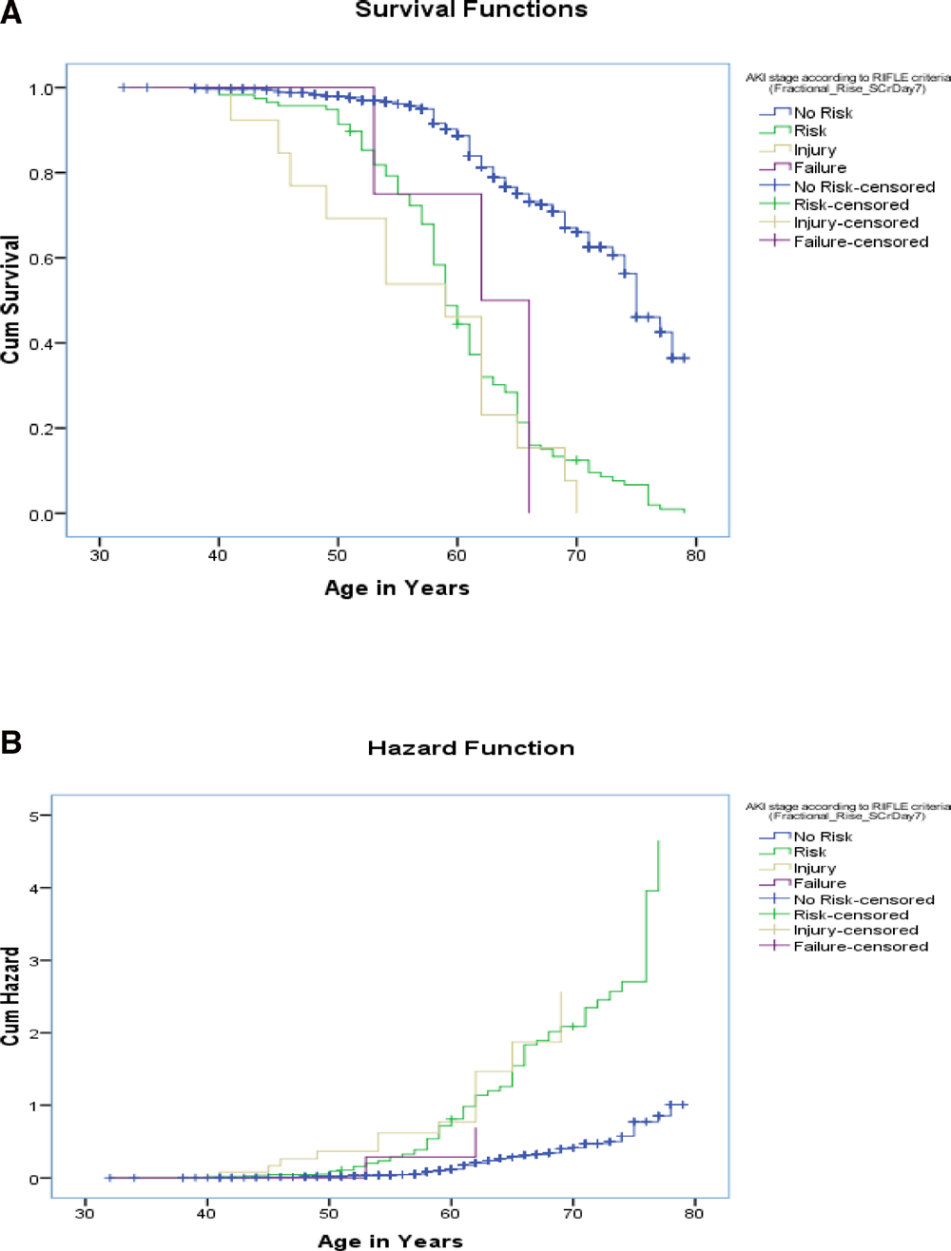
Test of equality of survival distributions (on age in yr) for the different levels of AKI stage according to risk, injury, failure, loss, end stage renal disease (RIFLE) criteria (Fractional_Rise_SCrDay7).

**Figure 9.**
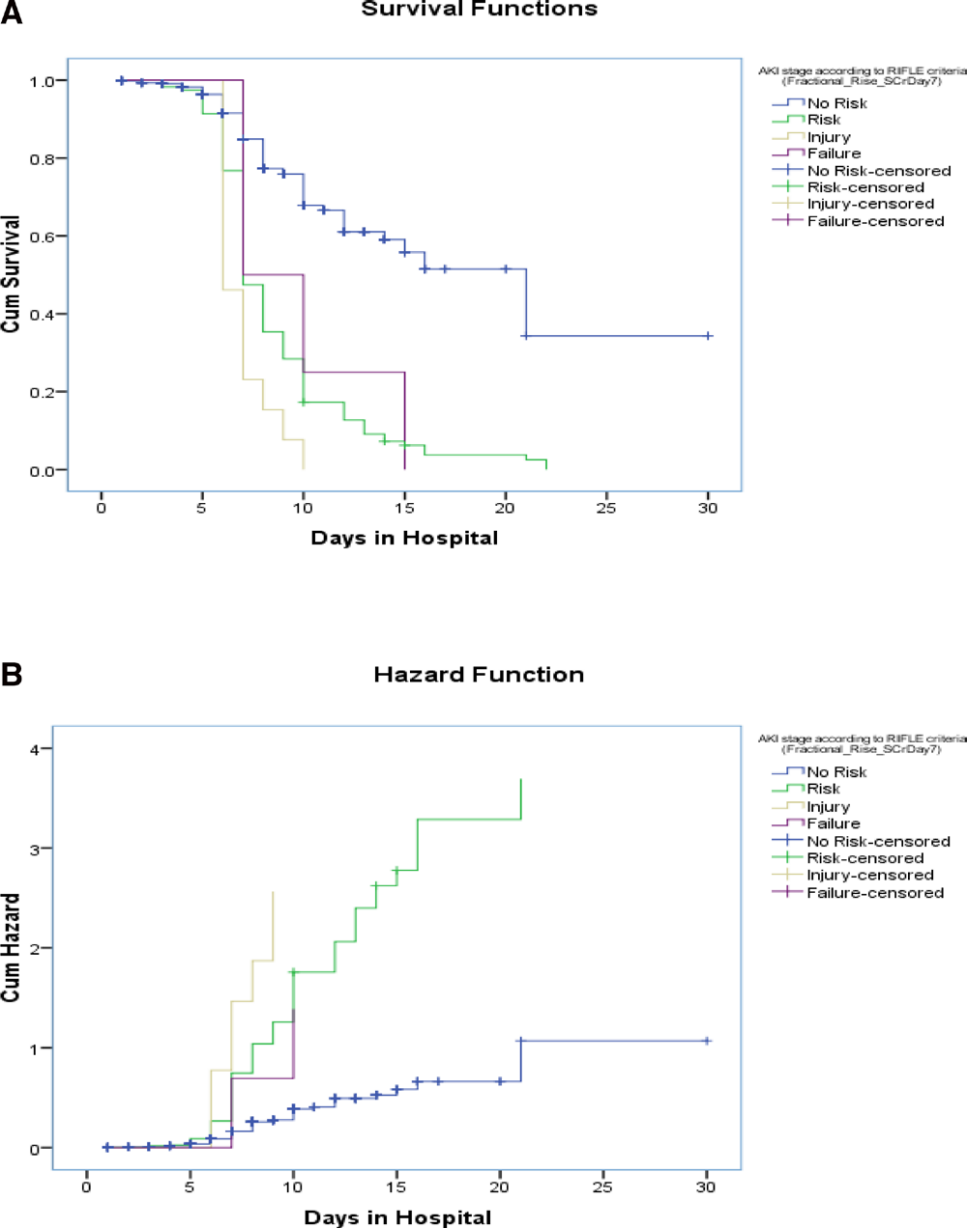
Test of equality of survival distributions (on d in hospital) for the different levels of acute kidney injury (AKI) stage according to risk, injury, failure, loss, end stage renal disease (RIFLE) criteria (Fractional_Rise_SCrDay7).

**Figure 10.**
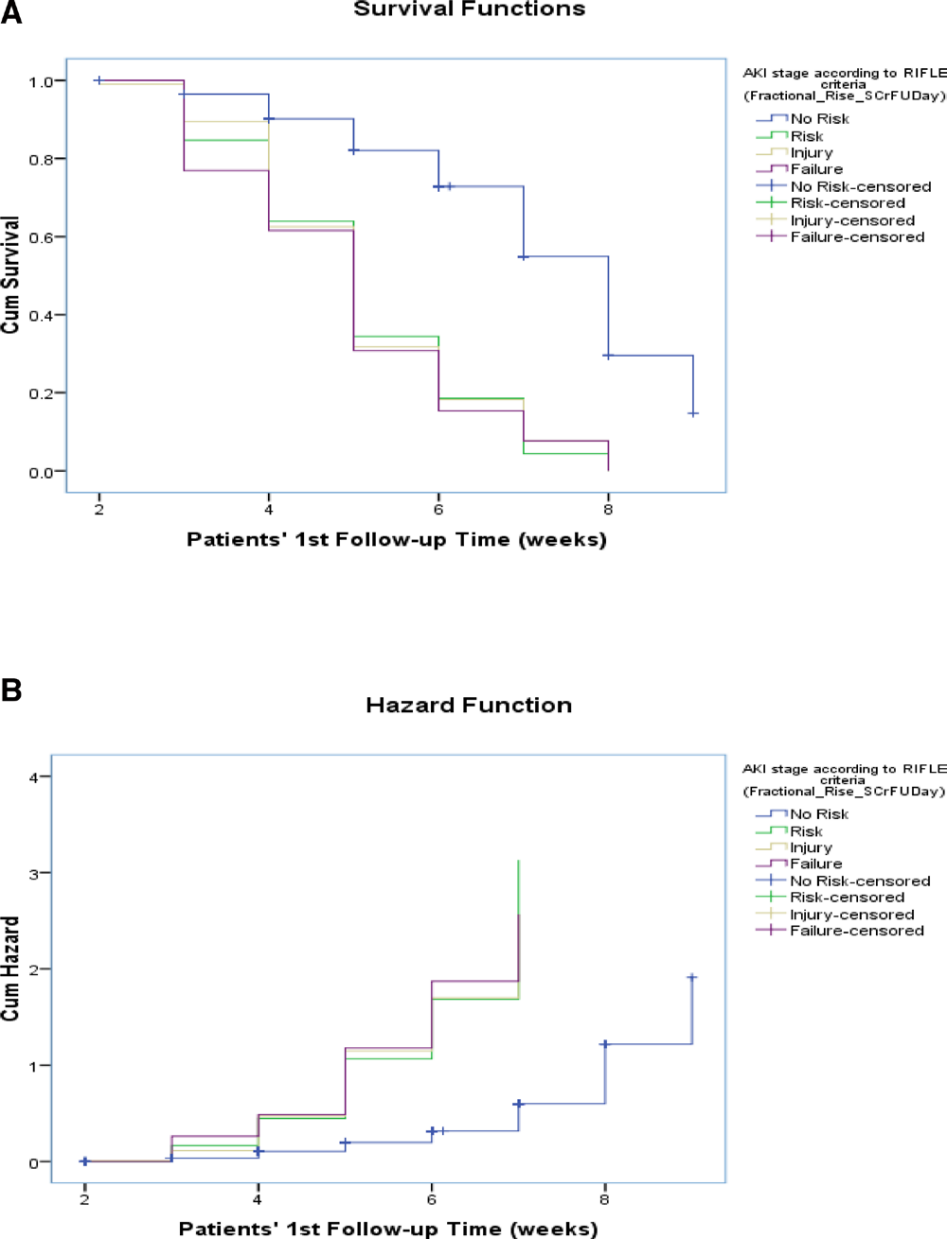
Test of equality of survival distributions (on follow-up time) for the different levels of acute kidney injury (AKI) stage according to risk, injury, failure, loss, end stage renal disease (RIFLE) criteria (Fractional Rise in S.Cr [serum creatinine] on follow-up d).

**Figure 11.**
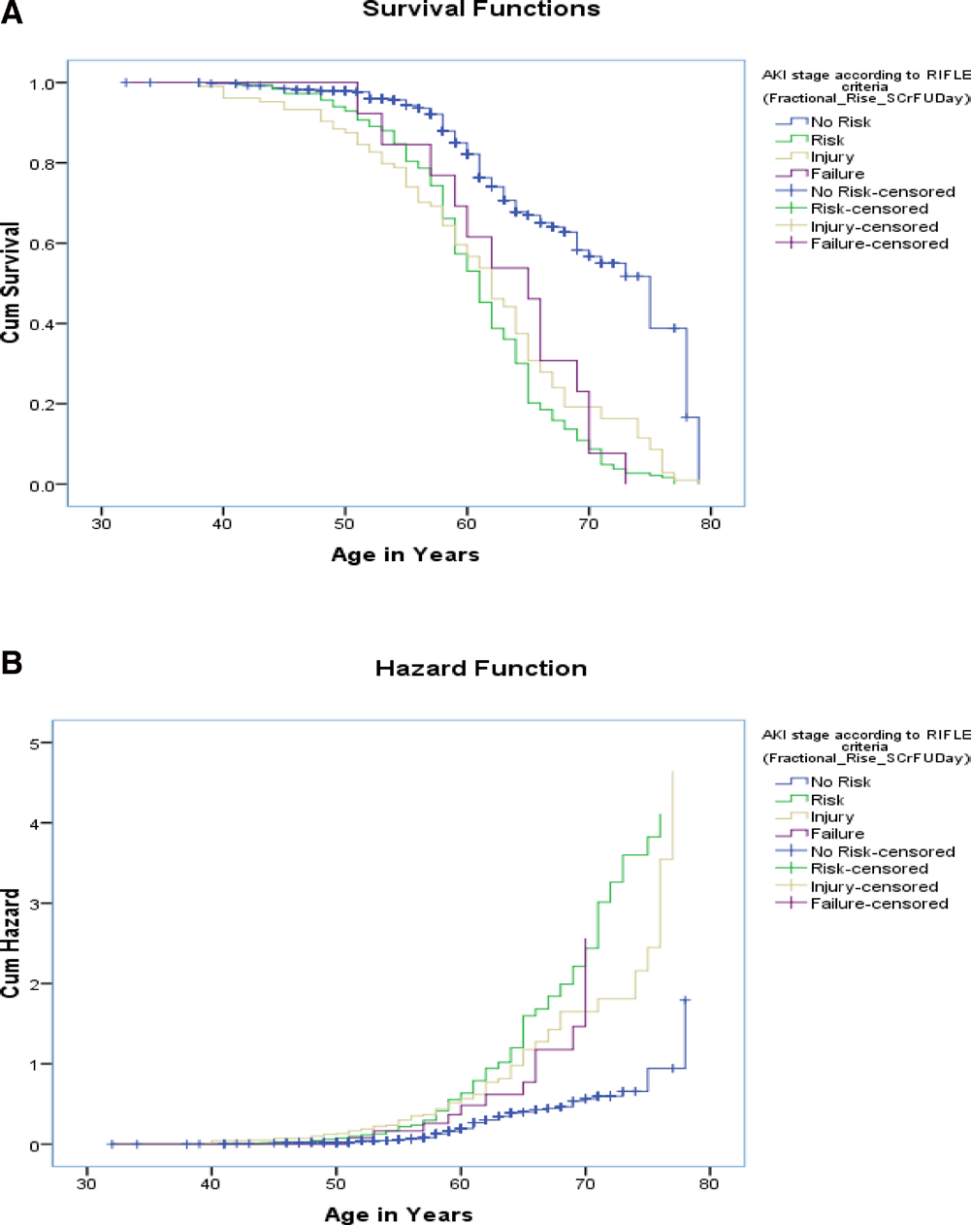
Test of equality of survival distributions (on age in yr) for the different levels of acute kidney injury (AKI) stage according to risk, injury, failure, loss, end stage renal disease (RIFLE) criteria (Fractional Rise in S.Cr [serum creatinine] on follow-up d).

Kaplan–Meier curve: Kaplan–Meier Curves were generated for AKI events that occurred on day 2 after surgery, day 7 after surgery, and renal injury at day 7 and on the follow-up day, stratified by eGFR levels (log rank values to show association). Significant associations were observed between the 2 groups (Figs. 5–11).

Laboratory tests: S.Cr levels showed a marked increase from the day of surgery to the second day after surgery in the paired-sample *t* test. The mean and standard deviation increased from 1.064 ± 0.2504 to 1.255 ± 0.2673 for baseline laboratory tests and tests, respectively, on day 2 of surgery (*P* = .000) (Supplementary Table 8, http://links.lww.com/MD/K166).

Assessment of renal function: the renal function is assessed by comparing the AKI prevalence at 3 points of time and these are compared with each other. The association between the each step is determined through chi-square test. The stages in loss of renal function/renal damage (Normal, Mild loss, Mild-Moderate loss, Moderate-Severe loss, Severe loss and Failure) were determined by criteria given at kidney.org (see Supplementary Table 4, http://links.lww.com/MD/K162). Renal Function changes/renal damage was measured using the values of percentage decrease in eGFR (Tables 2 and 3).

**Table 2:** Association of renal damage to demographic, pre-clinical and clinical variable.

Variables		Normal	Mild	Mild-moderate	Moderate-severe	Severe	*P* value
Day of surgery							
^1^Age	<60	65 (16.9%)	266 (69%)	44 (11.5%)	7 (1.8%)	3 (0.8%)	.030
	>60	42 (13.2%)	211 (66.1%)	60 (18.8%)	6 (1.9%)	0 (0%)	
^2^EF	<55 %	41 (10.3%)	268 (67.5%)	75 (18.9%)	10 (2.5%)	3 (0.8%)	.000
	>55 %	66 (21.5%)	209 (68%)	29 (9.5%)	3 (1.0%)	0 (0%)	
^3^IHD	Present	81 (16.1%)	336 (66.8%)	80 (16%)	3 (0.6%)	3 (0.6%)	.001
	Absent	26 (13%)	141 (70.1%)	24 (11.9%)	10 (5%)	0 (0%)	
^4^CKD	Present	4 (9.8%)	24 (58.5%)	10 (24.4%)	2 (4.9%)	1 (2.4%)	.034
	Absent	103 (15.5%)	453 (68.3%)	94 (14.2%)	11 (1.7%)	2 (0.3%)	
Post-surgical d 2							
Age	<60	39 (10.1%)	204 (53%)	100 (26%)	42 (10.9%)	0 (0%)	.000
	>60	11 (3.4%)	142 (44.5%)	118 (37%)	48 (15.1%)	0 (0%)	
EF	<55 %	17 (4.3%)	160 (40.3%)	142 (35.8%)	78 (19.6%)	0 (0%)	.000
	>55 %	33 (10.7%)	186 (60.6%)	76 (24.8%)	12 (3.9%)	0 (0%)	
^5^BMI	<27	15 (4.4%)	175 (51.5%)	101 (29.7%)	49 (14.4%)	0 (0%)	.028
	>27	35 (9.6%)	171 (47%)	117 (32.1%)	41 (11.3%)	0 (0%)	
CKD	Present	0 (0%)	17 (41.5%)	11 (26.8%)	13 (31.7%)	0 (0%)	.001
	Absent	50 (7.6%)	329 (49.6%)	207 (31.2%)	77 (11.6%)	0 (0%)	
Post-surgical d 7							
Gender	Male	30 (5.0%)	243 (41%)	202 (34.0%)	109 (18.3%)	10 (1.7%)	.030
	Female	8 (7.3%)	58 (52.7%)	24 (21.8%)	16 (14.5%)	4 (3.7%)	
Age	<60	24 (6.2%)	181 (47%)	119 (30.9%)	53 (13.8%)	8 (2.1%)	.013
	>60	14 (4.4%)	120 (37.6%)	107 (33.5%)	72 (22.6%)	6 (1.9%)	
EF	<55 %	16 (4.0%)	93 (23.4%)	168 (42.3%)	107 (27%)	13 (3.3%)	.000
	>55 %	22 (7.2%)	208 (67.7%)	58 (18.9%)	18 (5.9%)	1 (0.3%)	
^6^DM	Present	26 (6.3%)	183 (44.5%)	112 (27.3%)	79 (19.2%)	11 (2.7%)	.011
	Absent	12 (4.1%)	118 (40.3%)	114 (38.9%)	46 (15.7%)	3 (1.0%)	
Follow-up d							
Gender	Male	55 (9.3%)	152 (25.6%)	131 (22.1%)	219 (36.8%)	37 (6.2%)	.013
	Female	4 (3.6%)	41 (37.3%)	28 (25.5%)	28 (25.5%)	9 (8.1%)	
Age (yr)	<60	46 (12%)	125 (32.5%)	96 (25%)	106 (27.5%)	12 (3%)	.000
	>60	13 (4.1%)	68 (21.3%)	63 (19.8%)	141 (44.1%)	34 (10.7%)	
CKD	Present	2 (4.9%)	11 (26.8%)	12 (29.3%)	9 (21.9%)	7 (17.1%)	.026
	Absent	57 (8.6%)	182 (27.4%)	147 (22.2%)	238 (35.9%)	39 (5.9%)	
DM	Present	39 (9.5%)	118 (28.7%)	80 (19.5%)	141 (34.3%)	33 (8.0%)	.043
	Absent	20 (6.8%)	75 (25.6%)	79 (27%)	106 (36.2%)	13 (4.4%)	
Asthma	Present	0 (0%)	2 (20%)	0 (0%)	8 (80%)	0 (0%)	.043
	Absent	59 (8.5%)	191 (27.5%)	159 (23%)	239 (34.4%)	46 (6.6%)	
^7^AC (min)	<37	28 (7.7%)	110 (30.4%)	68 (18.8%)	137 (37.8%)	19 (5.3%)	.026
	>37	31 (9.0%)	83 (24.3%)	91 (26.6%)	110 (32.2%)	27 (7.9%)	
^8^CPB (min)	<58	20 (5.3%)	110 (29.3%)	82 (21.9%)	136 (36.3%)	27 (7.2%)	.026
	>58	39 (11.9%)	83 (25.2%)	77 (23.4%)	111 (33.7%)	19 (5.8%)	

○ Age in yr^1^.

○ LVEF: left ventricular ejection fraction (% age)^2^.

○ IHD: ischemic heart disease^3^.

○ CKD: chronic kidney disease^4^.

○ BMI: body mass index (kg/m^2^)^5^.

○ DM: diabetes mellitus^6^.

○ AC: aortic cross clamp time (min)^7^.

○ CPB: cardiopulmonary bypass time (min)^8^.

○ The stages in loss of renal function/renal damage (Normal, Mild loss, Mild-Moderate loss, Moderate-Severe loss, Severe loss and Failure) were determined by criteria given at kidney.org (see Supplementary Table 4)

○ Renal Function changes/renal damage was measured using the values of percentage decrease in eGFR

○ *P* values were determined by chi-square test.

○ In above table only significant results are tabulated. Detailed statistics are given in Supplementary material Table 6

**Table 3:** AKI stage versus pre-operative renal function loss/renal damage (2*2 contingency table).

		AKI d 7					
		No AKI (468)					AKI (236)
AKI d 2	No AKI (549)	409					140
	AKI (155)	59					96
		AKI Follow-up d					
		No AKI (310)					AKI (394)
AKI d 2	No AKI (549)	260					289
	AKI (155)	50					105
The association between renal function loss at d of surgery with AKI stage RIFLE criteria							
	Renal Function Loss/Renal Damage at the Day of Surgery	AKI stage according to RIFLE criteria (eGFR Decline at post-surgical d 2)					Total
		No risk		Risk			
	Normal	54		53			107
	Mild loss	341		136			477
	Mild-moderate loss	100		4			104
	Moderate-severe loss	13		0			13
	Severe loss	3		0			3
*P* = .000							
		AKI stage according to RIFLE criteria (eGFR Decline at post-surgical d 7)					
		No Risk	Risk			Injury	
	Normal	38	61			8	107
	Mild loss	293	176			8	477
	Mild-moderate loss	73	31			0	104
	Moderate-severe loss	5	8			0	13
	Severe loss	3	0			0	3
*P* = .000							
		AKI stage according to RIFLE criteria (eGFR Decline at Follow-up Visit)					
		No Risk	Risk		Injury	Failure	
	Normal	32	30		43	2	107
	Mild loss	196	185		96	0	477
	Mild-moderate loss	55	46		3	0	104
	Moderate-severe loss	7	6		0	0	13
	Severe loss	3	0		0	0	3
*P* = .000							

The baseline eGFR value was used to determine pre-operative renal function loss/renal damage. AKI staging was done using RIFLE criteria and association of both was determined using chi-square test.

AKI = acute kidney injury, eGFR = estimated glomerular filtration rate, RIFLE = risk, injury, failure, loss, end stage renal disease.

*P* values were determined by chi-square test.

Binary logistic regression: Variables with a *P* value < .2 were further explored through logistic regression analysis (Table 4). Binary logistic regression analysis confirmed an association between the duration of hospital stay (*P* = .035, OR = 1.42) and ejection fraction (*P* = .000, OR = 0.411) on day 7 after surgery. Among perioperative factors, CPB time (*P* = .032; OR = 0.721) was associated with AKI on follow-up day, and duration of hospital stay (*P* = .005; OR = 1.58) and age (*P* = .000; OR = 2.00) were associated with the development of AKI on follow-up after surgery. In multinomial logistic regression, AKI on day 7 was adjusted for age (*P* < .000, aOR = 2.460, CI = 1.761–3.435 for ejection fraction, *P* = .045, aOR = 0.715, CI = 0.516–0.992 for hospital stay).

**Table 4:** Regression analysis.

Binary logistic regression								
Acute kidney injury (AKI) prevalence on d 2 following surgery								
Variable			OR				Sig. (*P* value)	
Diabetes mellitus			1.287				.167	
Ejection fraction			0.720				.079	
Cerebrovascular accident			2.244				.078	
Acute kidney injury (AKI) prevalence on seventh d following surgery								
Variable		OR			Confidence interval (CI)			Sig.
Diagnosis		0.755			0.544–1.049			0.094
Age		1.262			0.922–1.727			0.146
Hospital stay		1.420			1.025–1.967			0.035
Ejection fraction		0.411			0.295–0.573			0.000
Acute kidney injury (AKI) prevalence on follow-up d								
Gender		0.720			0.479–1.083			0.115
Age		2.005			1.478–2.720			0.000
Diagnosis		0.804			0.587–1.102			0.176
Hospital stay		1.585			1.151–2.181			0.005
Diabetes mellitus		1.269			0.937–1.719			0.123
CPB-time		0.721			0.535–0.972			0.032
Multinomial logistic regression analysis								
Variable	Sig.		OR	CI		Sig.	aOR	CI
Acute kidney injury (AKI) prevalence on seventh d following surgery. (Ejection Fraction and Hospital Stay adjusted for Age)								
Hospital stay	0.035		1.420	1.025–1.967		0.045	0.715	0.516–0.992
Ejection fraction	0.000		0.411	0.295–0.573		0.000	2.460	1.761–3.435
Acute kidney injury (AKI) prevalence on follow-up d. (Age adjusted for Ejection Fraction, Hospital Stay and CPB time was adjusted for Age)								
Age	0.000		2.005	1.478–2.720		0.000	0.497	0.367–0.675
Hospital stay	0.005		1.585	1.151–2.181		0.011	1.522	1.101–2.105
CPB-time	0.032		0.721	0.535–0.972		0.020	0.696	0.514–0.944

aOR = adjusted odds ratio, CI = confidence interval (95%), CPB-time = cardiopulmonary bypass time, OR = odds ratio.

## 4. Discussion

Prevalence of AKI: on the second day after CABG surgery, 155 AKI cases were identified. On the seventh day following surgery and on the follow-up day, the prevalence of AKI in the present study was fairly high. A comparative analysis of the prevalence of AKI on the second and seventh post-operative days was performed to determine the progress of AKI. Of 155 patients who developed AKI on the second post-operative day, 96 patients remained in the AKI group until the seventh day. On the seventh day, 140 new cases were included in the AKI group. On the follow-up day, 289 new cases of AKI were observed, with a total of 394 cases recorded during follow-up. This comparison indicated that an acute surge of AKI occurred on the second day following surgery, but the number of AKI cases continued to increase on day 7 and the follow-up day.

The assessment of AKI stages was again performed on the seventh day, 276 risk assessments were performed, and 16 patients with renal injury were identified. On the follow-up day, there were 142 injuries and 2 cases of acute renal failure (based on eGFR values). S.Cr values predicted renal failure in 3 patients on day 2, 4 patients on day 7, and 13 patients on the follow-up day. Analysis of AKI stages on the second post-operative day revealed no injury. Fortunately, no injury or failure was predicted with eGFR values on day 2 and no failure on day 7.

Early detection of risk through assessment of pre-operative renal function can prevent adverse outcomes in patients undergoing surgery. Cases identified as high-risk on the second day following surgery should be scheduled by their primary healthcare provider for frequent follow-up. Therefore, they can be carefully assessed for the emergence of any renal injury and treated with RRT if appropriate. Early intervention through dialysis can prevent permanent kidney damage in cases of acute renal failure.

Associations: AKI can predict mortality.^[17]^ In our study, age >60 years (follow-up day *P* = .000), LVEF (seventh day *P* < .000), and Days stayed in hospital (day 7 *P* < .035; follow-up, *P* < .005) were risk factors for the development of AKI.

Diabetes along with angina pectoris^[20]^ and diabetes alone,^[21,22]^ mean age change, S.Cr, and HTN^[22]^ are mentioned as risk factors in the literature. Pump surgery, transfusions, prolonged mechanical ventilation,^[23]^ peripheral artery disease, pre-operative renal function, and sex have been identified as risk factors. The type and length of surgery, damaged vessels, left main disease, statins, RBC infusion, pre-operative cardiac problems, and reoperation for bleeding were co-occurring variables.^[24]^ Poor EF, intra-aortic balloon pump insertion, and extended CPB duration are independent risk factors for developing ARF necessitating dialysis after CABG.^[25]^

Sex association as a risk factor has not been reported in the present study, as in some previous studies.^[26]^ AKI led to longer median hospital stays and stays in the critical care unit, as well as an 8-fold increase in 30-day mortality.^[27]^ BMI > 40 kg/m^2[28]^ and chronic hyperglycemia (HbA1c ≥ 6.0%) were risk factors. Coexisting risk factors include age, compromised kidney function, pre-operative atrial AF, and duration of surgery.^[29]^ The interval between cardiac catheterization and surgery, elevated post-operative C-reactive protein levels, and erythrocyte sedimentation rate are independent predictors of acute kidney damage in patients undergoing isolated coronary artery bypass grafting.^[30]^ The risk factors exaggerate the incidence of AKI.^[31]^

The present study explored risk factors that may lead to the development of AKI. Binary logistic regression analysis for this population data set confirmed the association between the duration of hospital stay and ejection fraction and the development of AKI on day 7 after surgery. Among perioperative factors, CPB time was associated with AKI on the follow-up day, and the duration of hospital stay and age were associated with the development of AKI on the day of follow-up after surgery. In multinomial logistic regression, AKI on day 7 was adjusted for age, ejection fraction, and hospital stay. The odds ratio for hospital stay changed from 1.42 to 0.715 when adjusted for age, indicating that age affects the duration of stay at the hospital.

LVEF along with COPD has been previously reported.^[32]^ Acute MI^[20]^ and urgent surgery^[33]^ were also identified, in addition to the risk factors mentioned above. A positive association between AC time and AKI has been reported earlier, along with other risk factors such as CPB, anemia, and CKD low cardiac output syndrome.^[34]^ CPB time was identified as an important risk factor in the present analysis, both in binary and multinomial logistic regression (*P* = .032, OR = 0.721). When we adjusted CPB time with age, a positive association was observed, which means that if CPB time is longer in elderly patients (>60 years), the chances that the prevalence of AKI will be higher are higher.

Assessment of renal functional changes: AKI and CKD have a complex interaction, since CKD increases the risk of AKI, while AKI itself can cause it.^[5]^ High levels of post-operative S.Cr values and AKI can predict the risk of developing CKD^[35]^ 1% to 1.7% of whom will need dialysis^[32]^ and 30-day mortality following cardiac and thoracic aortic surgery.^[13]^ In Patients with a mildly reduced baseline eGFR by improving cardiac function.^[21]^

The pre-operative renal function and efficacy determine the stage of AKI that will be detected post-operatively. S.Cr levels may underestimate the severity of kidney damage; therefore, we assessed renal function on the basis of eGFR values according to the standard protocol.^[36]^ The trend in renal function change was observed on days 2, 7, and on follow-up days. In the present study, 2 patients developed acute renal failure and were identified on the first follow-up visit. They were referred to the nephrology department for further evaluation and early intervention if needed. Renal function must be protected from dehydration. In such cases, early renal replacement treatment should be considered. Renal Replacement Treatment (RRT) must be implemented in the ICU or CCU, and the patient must be cared for by highly qualified professionals during the post-CABG phase.

Renal function was poor among women, with 3.7% of women having severe loss of renal function compared with men (1.7%), as determined by staging performed on day 7. A similar sex trend was observed on the follow-up day. In this study population, renal function declined at the same rate in the above- and below-sixty-year-old groups until day 7 of surgery (60/>60 = 2.1/1.9). A remarkable change was observed at the follow-up visit, where 10.7% of the patient age of sixty developed severe renal loss compared to 3% in the below-the-age group. A very high number of patients (44.1%) developed moderate-to-high levels of renal function loss. This finding can lead to the assumption that the elderly are more at risk of loss of renal function, and this age group must be considered while performing pre-operative assessment (also see Supplementary Table 9, http://links.lww.com/MD/K167).

In the present cohort, 27% of the patients with EF <55% developed moderate to severe and 3.3% developed severe loss of renal function at day 7 after surgery, which is remarkably higher than the group of patients with EF >55% (0.3%). Patients with a body mass index <27 kg/m^2^ developed moderate-to-severe loss of kidney function (14.4%) on the second day following surgery. No severe loss of kidney function was observed in any of the BMI groups.

Patients with preexisting CKD must be carefully monitored for adverse outcomes as they already have compromised renal function. In our cohort, there were 41 patients with CKD, of whom 21.9% had developed a moderate to severe loss, while 17.1% had developed a severe loss of renal function when they were observed on the follow-up day (Supplementary Table 10, http://links.lww.com/MD/K168). Asthma, AC, and CPB time affect renal function in the long term, but not immediately. DM is a high-risk comorbidity for renal function. On the seventh day after surgery, renal function assessment was performed; 79 diabetic patients (19.2%) had moderate to severe loss of renal function, and 2.7% (n = 11) had severe loss of renal function. Renal function decreased further, and follow-up assessment revealed that 141 (34.3%) diabetic patients developed moderate to severe kidney disease, while 33 (8.0%) developed severe kidney disease.

AKI detection in ordinary clinical practice appears to be low, particularly at lower stages of AKI.^[37]^ Angiotensin-converting enzyme inhibitors and angiotensin receptor blockers should be stopped before surgery, and nephrotoxins should be avoided to lower this risk. Goal-directed therapy with personalized hemodynamic and fluid control, the use of circuits with biocompatible coatings, the implementation of minimally invasive extracorporeal circulation, and lung protective ventilation are some of the intraoperative preventative measures^[38]^ given in the kidney disease improving global outcomes bundle.^[39]^ The risk of acute renal injury can be predicted using already accessible hospital data. Doctors and surgeons can focus on high-risk groups using their knowledge of the pre-operative risk factors. Measures should be taken to lower the risk of severe complications following surgery.

## 5. Conclusion

AKI can cause increased early mortality and has an impact on the long-term outcomes of surgery and patient survival. In order to prevent surgery outcomes from being impacted, RRT should be initiated as soon as possible in patients who experience AKI following CABG. For children, peritoneal dialysis can be used in children. Every population has different patient characteristics, suggesting that multicenter observational and interventional studies on acute renal injury are necessary. Interventions should be implemented to reduce the pre-operative factors associated with post-operative complications.

## 6. Limitations and recommendation

The purpose of this study was to obtain as many details as possible from hospital records, but there were still missing values, so we had to exclude those entries, thus making the sample slightly smaller. Follow-up visit data were absent, and we could not predict early and late mortality associated with AKI. Fewer risk factors were identified than in previous studies.

○ High-risk groups must be identified prior to surgery.
○ As AKI is a common complication, early RRT should be considered.
○ There must be follow-up to evaluate the success of surgery and study the effect of AKI on early and late mortality.

## Acknowledgments

Direct technical help in the form of statistics/data manipulation was provided by Muhammad Ali Raza, Data analyst consultant, UNICEF Pakistan, muhammadalirazaeco@gmail.com. Google Scholar, PubMed, and Science Direct provided indirect assistance along with SciFinder® and Sci-hub for literature review.

## Author contributions

**Conceptualization:** Awais Ahmad Nizami, Khawar Sultan.

**Data curation:** Adnan Mushtaq, Amna Akbar.

**Formal analysis:** Khawar Sultan, Naeem Ullah, Qudsia Umaira Khan.

**Methodology:** Rukhsana Manzoor, Humayun Saleem.

**Resources:** Sarosh Alvi.

**Software:** Sabahat Tasneem.

**Supervision:** Muhammad Sajid Rafiq Abbasi, Mohammad Saleem Khan.

**Writing – original draft:** Muhammad Sajid Rafiq Abbasi, Mohammad Saleem Khan.

**Writing – review & editing:** Sarosh Alvi, Awais Ahmad Nizami, Sarosh Khan Jadoon.

## Supplementary Material





















## References

[1] Bachar BJ Manna B. Coronary Artery Bypass Graft. In Treasure Island (FL); 2023. Available from: https://pubmed.ncbi.nlm.nih.gov/29939613/

[2] Spadaccio C Benedetto U. Coronary artery bypass grafting (CABG) vs. percutaneous coronary intervention (PCI) in the treatment of multivessel coronary disease: quo vadis? - A review of the evidences on coronary artery disease. Ann Cardiothorac Surg. 2018;7:506–15. 30094215 10.21037/acs.2018.05.17 PMC6082779

[3] Mehta RL Kellum JA Shah SV. Acute Kidney Injury Network: report of an initiative to improve outcomes in acute kidney injury. Critical care (London, England) 2007;11:R31. 17331245 10.1186/cc5713 PMC2206446

[4] Olsson D Sartipy U Braunschweig F. Acute kidney injury following coronary artery bypass surgery and long-term risk of heart failure. Circ Heart Fail 2013;6:83–90. 23230310 10.1161/CIRCHEARTFAILURE.112.971705

[5] Bedford M Farmer C Levin A. Acute kidney injury and CKD: chicken or egg? Am J Kidney Dis 2012;59:485–91. 22444492 10.1053/j.ajkd.2011.09.010

[6] Serraino GF Provenzano M Jiritano F. Risk factors for acute kidney injury and mortality in high risk patients undergoing cardiac surgery. PLoS One. 2021;16:e0252209. 34019579 10.1371/journal.pone.0252209 PMC8139497

[7] Rosner MH Okusa MD. Acute kidney injury associated with cardiac surgery. Clin J Am Soc Nephrol. 2006;1:19–32. 17699187 10.2215/CJN.00240605

[8] Herget-Rosenthal S Marggraf G Hüsing J. Early detection of acute renal failure by serum cystatin C. Kidney Int. 2004;66:1115–22. 15327406 10.1111/j.1523-1755.2004.00861.x

[9] Demirjian S Bashour CA Shaw A. Predictive accuracy of a perioperative laboratory test–based prediction model for moderate to severe acute kidney injury after cardiac surgery. JAMA. 2022;327:956–64. 35258532 10.1001/jama.2022.1751 PMC8905398

[10] Ryckwaert F Boccara G Frappier J-M. Incidence, risk factors, and prognosis of a moderate increase in plasma creatinine early after cardiac surgery. Crit Care Med. 2002;30:1495–8. 12130968 10.1097/00003246-200207000-00016

[11] Lin C-Y Chen Y-C. Acute kidney injury classification: AKIN and RIFLE criteria in critical patients. World J Crit care Med. 2012;1:40–5. 24701400 10.5492/wjccm.v1.i2.40 PMC3953858

[12] Levey AS Levin A Kellum JA. Definition and classification of kidney diseases. Am J Kidney Dis 2013;61:686–8. 23582249 10.1053/j.ajkd.2013.03.003

[13] Lassnigg A Schmidlin D Mouhieddine M. Minimal changes of serum creatinine predict prognosis in patients after cardiothoracic surgery: a prospective cohort study. J Am Soc Nephrol. 2004;15:1597–605. 15153571 10.1097/01.asn.0000130340.93930.dd

[14] Lassnigg A Schmid ER Hiesmayr M. Impact of minimal increases in serum creatinine on outcome in patients after cardiothoracic surgery: do we have to revise current definitions of acute renal failure? Crit Care Med. 2008;36:1129–37. 18379238 10.1097/CCM.0b013e318169181a

[15] Liotta M Olsson D Sartipy U. Minimal changes in postoperative creatinine values and early and late mortality and cardiovascular events after coronary artery bypass grafting. Am J Cardiol. 2014;113:70–5. 24176074 10.1016/j.amjcard.2013.09.012

[16] Rydén L Ahnve S Bell M. Acute kidney injury following coronary artery bypass grafting: early mortality and postoperative complications. Scand Cardiovasc J. 2012;46:114–20. cited2023Feb16. 22324648 10.3109/14017431.2012.657229

[17] Khwaja A. KDIGO clinical practice guidelines for acute kidney injury. Nephron Clin Pract. 2012;120:c179–84. 22890468 10.1159/000339789

[18] Historical eGFR Calculator - NIDDK.

[19] Moguel-González B Wasung-de-Lay M Tella-Vega P. Acute kidney injury in cardiac surgery. Rev Invest Clin 2013;65:467–75. 24687353

[20] Al-Githmi IS Abdulqader AA Alotaibi A. Acute kidney injury after open heart surgery. Cureus. 2022;14:10–5. 10.7759/cureus.25899 PMC9278797 35844317

[21] Jiarui Xu MD Chen MSc X Yeqing Xie MD. Improvement of cardiac function after coronary artery bypass grafting surgery reduces the risk of postoperative acute kidney injury. Clin Cardiol. 2022;45:173–9. 35094407 10.1002/clc.23785 PMC8860479

[22] Ng RRG Chew STH Liu W. Identification of modifiable risk factors for acute kidney injury after coronary artery bypass graft surgery in an Asian population. J Thorac Cardiovasc Surg. 2014;147:1356–61. 24183907 10.1016/j.jtcvs.2013.09.040

[23] Mirmohammad-Sadeghi M Naghiloo A Najarzadegan MR. Evaluating the relative frequency and predicting factors of acute renal failure following coronary artery bypass grafting. ARYA Atheroscler. 2013;9:287–92. 24302937 PMC3845695

[24] Kang W Wu X. Pre-, intra-, and post-operative factors for kidney injury of patients underwent cardiac surgery: a retrospective cohort study. Med Sci Monit 2019;25:5841–9. 31383840 10.12659/MSM.915996 PMC6693368

[25] Zhou JY Liu XC Yang Q. Risk factors for development of acute renal failure in 5077 coronary artery bypass grafting patients in the current era. J Card Surg. 2022;37:4891–8. 36378933 10.1111/jocs.17164

[26] Bell J Sartipy U Holzmann MJ. The association between acute kidney injury and mortality after coronary artery bypass grafting was similar in women and men. J Cardiothorac Vasc Anesth. 2022;36:962–70. 34969562 10.1053/j.jvca.2021.11.036

[27] Fisher L-A Stephenson S Reid MT. Acute kidney injury following cardiopulmonary bypass in Jamaica. JTCVS Open 2022;11:161–75. 36172431 10.1016/j.xjon.2022.05.012 PMC9510884

[28] Pacholewicz J Kuligowska E Szylińska A. The rate of postoperative mortality and renal and respiratory complications are increased in patients with extreme obesity undergoing cardiac surgery – a Retrospective Observational Cohort Study of 8848 Patients. Diabetes, Metab Syndr Obes 2023;16:1155–66. 37122675 10.2147/DMSO.S400597 PMC10145505

[29] Oezkur M Wagner M Weismann D. Chronic hyperglycemia is associated with acute kidney injury in patients undergoing CABG surgery--a cohort study. BMC Cardiovasc Disord. 2015;15:41. 25964053 10.1186/s12872-015-0028-y PMC4443518

[30] Görür D Şaşkin H Düzyol C. What is the optimal time interval between heart catheterization and surgery to prevent acute kidney injury in patients with isolated coronary artery bypass? Postepy Kardiol Interwencyjnej 2022;18:137–45. 36051840 10.5114/aic.2022.118157 PMC9421509

[31] Amini S Najafi MN Karrari SP. Risk factors and outcome of acute kidney injury after isolated CABG surgery: a prospective cohort study. Braz J Cardiovasc Surg 2019;34:70–5. 30810677 10.21470/1678-9741-2017-0209 PMC6385837

[32] Eriksen BO Hoff KRS Solberg S. Prediction of acute renal failure after cardiac surgery: retrospective cross-validation of a clinical algorithm. Nephrol Dial Transplant 2003;18:77–81. 12480963 10.1093/ndt/18.1.77

[33] Axtell AL Fiedler AG Melnitchouk S. Correlation of cardiopulmonary bypass duration with acute renal failure after cardiac surgery. J Thorac Cardiovasc Surg. 2020;159:170–178.e2. 30826102 10.1016/j.jtcvs.2019.01.072

[34] Alhulaibi AA Alruwaili AM Alotaibi AS. Validation of various prediction scores for cardiac surgery-associated acute kidney injury. J Saudi Heart Assoc 2022;34:222–31. 36816793 10.37616/2212-5043.1322 PMC9930984

[35] Ishani A Nelson D Clothier B. The magnitude of acute serum creatinine increase after cardiac surgery and the risk of chronic kidney disease, progression of kidney disease, and death. Arch Intern Med. 2011;171:226–33. 21325112 10.1001/archinternmed.2010.514

[36] Bouma HR Mungroop HE de Geus AF. Acute kidney injury classification underestimates long-term mortality after cardiac valve operations. Ann Thorac Surg. 2018;106:92–8. 29501641 10.1016/j.athoracsur.2018.01.066

[37] Schanz M Schöffski O Kimmel M. Under-recognition of acute kidney injury after cardiac surgery in the ICU impedes early detection and prevention. Kidney Blood Press Res. 2021;47:50–60. 34775389 10.1159/000519536

[38] Ostermann M Weerapolchai K Lumlertgul N. Prevention of Acute Kidney Injury After Cardiac Surgery BT - Annual Update in Intensive Care and Emergency Medicine 2022. In: Vincent J-L, editor. Cham: Springer International Publishing; 2022. p. 223–34. Available from: 10.1007/978-3-030-93433-0_18

[39] Zarbock A Küllmar M Ostermann M. Prevention of cardiac surgery-associated acute kidney injury by implementing the KDIGO Guidelines in High-Risk Patients Identified by Biomarkers: the PrevAKI-Multicenter Randomized Controlled Trial. Anesth Analg. 2021;133:292–302. 33684086 10.1213/ANE.0000000000005458

