# Supplementary material for: Assessment of renal function and prevalence of acute kidney injury following coronary artery bypass graft surgery and associated risk factors: A retrospective cohort study at a tertiary care hospital in Islamabad, Pakistan
Source: Medicine (Baltimore). 2023 Oct 20;102(42):e35482. doi: 10.1097/MD.0000000000035482 (PMC10589541; doi:10.1097/MD.0000000000035482)
Supplement: Supplementary file 1 [file medi-102-e35482-s001.docx]

Supplementary Table 1: Division of continuous variables’ value into two categories (divided by median as a cut-off value)

| **Variable** | **Cut off value** | **Category 1** | **Category 2** |
| --- | --- | --- | --- |
| Age (years) | 60 | < 60 | > 60 |
| Days Stayed at Hospital (days) | 7 | < 7 | > 7 |
| Body Mass Index (kg/m^2^) | 27 | < 27 | > 27 |
| Ejection Fraction (percentage) | 55 | < 55 | > 55 |
| Aortic cross clamp time (minutes) | 37 | < 37 | > 37 |
| Cardiopulmonary bypass time (minutes) | 58 | < 58 | > 58 |
