# Supplementary material for: Assessment of renal function and prevalence of acute kidney injury following coronary artery bypass graft surgery and associated risk factors: A retrospective cohort study at a tertiary care hospital in Islamabad, Pakistan
Source: Medicine (Baltimore). 2023 Oct 20;102(42):e35482. doi: 10.1097/MD.0000000000035482 (PMC10589541; doi:10.1097/MD.0000000000035482)
Supplement: Supplementary file 2 [file medi-102-e35482-s002.docx]

Supplementary Table 2: Stages of AKI according to RIFLE criteria

| Risk of renal failure, injury , failure of kidney function, loss of kidney function, and end-stage renal failure | | |
| --- | --- | --- |
| RIFLE (an acute rise in S.Cr over 7d) | | |
| Risk | Increase in S.Cr ≥ 1.5 × baseline or decrease in GFR ≥ 25% | UO < 0.5 mL/kg per hour × 6 h |
| Injury | Increase in S.Cr ≥ 2.0 × baseline or decrease in GFR ≥ 50% | UO < 0.5 mL/kg per hour × 12 h |
| Failure | Increase in S.Cr ≥ 3.0 × baseline or S.Cr ≥ 4.0 mg/dL (354 mol/L) or decrease in GFR ≥ 75% | UO < 0.3 mL/kg per hour × 24 h or anuria × 12 h |
| Loss | Complete loss of kidney function more than 4 weeks |  |
| ESKD | Complete loss of kidney function more than 3 months | Same as RIFLE |
| Lin CY et al . RIFLE and AKIN criteria:  AKIN: Acute Kidney Injury Network; ESKD: End-stage kidney disease; GFR: Glomerular filtration rate; RIFLE: Risk of renal failure, injury to the kidney, failure of kidney function, loss of kidney function, and end-stage kidney disease; S.Cr: Serum creatinine; UO: Urine output. | | |
