# Supplementary material for: Assessment of renal function and prevalence of acute kidney injury following coronary artery bypass graft surgery and associated risk factors: A retrospective cohort study at a tertiary care hospital in Islamabad, Pakistan
Source: Medicine (Baltimore). 2023 Oct 20;102(42):e35482. doi: 10.1097/MD.0000000000035482 (PMC10589541; doi:10.1097/MD.0000000000035482)
Supplement: Supplementary file 3 [file medi-102-e35482-s003.docx]

Supplementary Table 3: AKIN staging

| AKI | Criteria |
| --- | --- |
| 0 | No AKI according to classification (*i.e.* increase in serum creatinine < 150% and <26.4 mmol/L (≥0.3 mg/dL)) |
| 1 | Increase in serum creatinine to 150–199% (1.5–1.99 x increased compared with baseline) OR increase of ≥26.4 mmol/L (≥0.3 mg/dL) |
| 2 | Increase in serum creatinine to 200–299% (2.0–2.99 × increase compared with baseline) |
| 3 | Increase in serum creatinine to ≥300% (3 × increase compared with baseline) OR serum creatinine of ≥354 μmol/L (≥4.0 mg/dL) with an acute increase of at least 44 μmol/L (0.5 mg/dL) |

The acute kidney injury network (AKIN) classification was used to classify AKI among patients in the dataset, using the perioperative change in serum creatinine.
