# Supplementary material for: Assessment of renal function and prevalence of acute kidney injury following coronary artery bypass graft surgery and associated risk factors: A retrospective cohort study at a tertiary care hospital in Islamabad, Pakistan
Source: Medicine (Baltimore). 2023 Oct 20;102(42):e35482. doi: 10.1097/MD.0000000000035482 (PMC10589541; doi:10.1097/MD.0000000000035482)
Supplement: Supplementary file 4 [file medi-102-e35482-s004.docx]

Supplementary Table 4: Stages of Renal Damage/ Loss of Renal Function

|  | Status | eGFR | Kidney Function |
| --- | --- | --- | --- |
| G1 | Probability of Renal Damage but with normal Renal Function | 90 or above | 90-100% |
| G2 | Renal Damage with mild loss of Renal function | 60-89 | 60-89% |
| G3a | Mild-moderate loss of Renal function | 45-59 | 45-59% |
| G3b | Moderate-severe loss of Renal function | 30-44 | 30-44% |
| G4 | Severe loss of Renal function | 15-29 | 15-29% |
| G5 | Kidney failure | Less than 15 | Less than 15% |
| [www.kidney.org](http://www.kidney.org) : https://www.kidney.org/professionals/explore-your-knowledge/how-to-classify-ckd | | | |
