# Supplementary material for: Assessment of renal function and prevalence of acute kidney injury following coronary artery bypass graft surgery and associated risk factors: A retrospective cohort study at a tertiary care hospital in Islamabad, Pakistan
Source: Medicine (Baltimore). 2023 Oct 20;102(42):e35482. doi: 10.1097/MD.0000000000035482 (PMC10589541; doi:10.1097/MD.0000000000035482)
Supplement: Supplementary file 5 [file medi-102-e35482-s005.docx]

Supplementary Table 5: Prevalence of AKI

| Variable | AKI at Day 2 | | AKI at Day 7 | | AKI at Follow-up Day | |
| --- | --- | --- | --- | --- | --- | --- |
| 704(100%) | AKI= 155 | No-AKI= 549 | AKI= 236 | No-AKI= 468 | AKI= 394 | No-AKI= 310 |
| Gender | | | | | | |
| Male | 127(81.9%) | 467 (85.1%) | 203 (86%) | 391 (83.5%) | 340(86.3%) | 254 (81.9%) |
| Female | 28 (18.1%) | 82 (14.9%) | 33 (14%) | 77 (16.5%) | 54 (13.7%) | 56 (18.1%) |
| Diagnosis | | | | | | |
| TVCAD | 100(64.5%) | 371 (67.6%) | 148(62.7%) | 323 (69.0%) | 272 (69%) | 199 (64.2%) |
| TVCAD+LMS | 55 (35.5%) | 178 (32.4%) | 88 (37.3%) | 145 (31.0%) | 122 (31%) | 111 (35.8%) |
| Hypertension (HTN) | | | | | | |
| Yes | 103(66.5%) | 374(68.1%) | 167(70.8%) | 310 (66.2%) | 271(68.8%) | 206 (66.5%) |
| No | 52 (33.5%) | 175 (31.9%) | 69 (29.2%) | 158(33.8%) | 123(31.2%) | 104(33.5%) |
| Diabetes Mellitus (DM) | | | | | | |
| Yes | 83 (53.5%) | 328 (59.7%) | 136(57.6%) | 275 (58.8%) | 220(55.8%) | 191 (61.6%) |
| No | 72 (46.5%) | 221 (40.3%) | 100(42.4%) | 193 (41.2%) | 174(44.2%) | 119 (38.4%) |
| Ischemic Heart Disease (IHD) | | | | | | |
| Yes | 109(70.3%) | 394 (71.8%) | 164(69.5%) | 339 (72.4%) | 280(71.1%) | 223 (71.9%) |
| No | 46 (29.7%) | 155 (28.2%) | 72 (30.5%) | 129 (27.6%) | 114 (28.9%) | 87 (28.1%) |
| Chronic Kidney Disease (CKD) | | | | | | |
| Yes | 7 (4.5%) | 34 (6.2%) | 13 (5.5%) | 28 (6%) | 17 (4.3%) | 24 (7.7%) |
| No | 148(95.5%) | 515 (93.8%) | 223(94.5%) | 440 (94%) | 377(95.7%) | 286 (92.3%) |
| Asthma | | | | | | |
| Yes | 1 (0.6%) | 9 (1.6%) | 3 (1.3%) | 7 (1.5%) | 7 (1.8%) | 3 (1%) |
| No | 154(99.4%) | 540 (98.4%) | 233(98.7%) | 461 (98.5%) | 387(98.2%) | 307 (99%) |
| Smoking | | | | | | |
| Yes | 69 (44.5%) | 254 (46.3%) | 103 (43.6%) | 220 (47%) | 178 (45.2%) | 145 (46.8%) |
| No | 86 (55.5%) | 295 (53.7%) | 133 (56.4%) | 248 (53%) | 216 (54.8%) | 165 (53.2%) |
| Cerebro Vascular Accident (CVA) | | | | | | |
| Yes | 8 (5.2%) | 13 (2.4%) | 8 (3.4%) | 13 (2.8%) | 11 (2.8%) | 10 (3.2%) |
| No | 147(94.8%) | 536 (97.6%) | 228(96.6%) | 455 (97.2%) | 383(97.2%) | 300 (96.8%) |
| Body Mass Index (kg/m2) | | | | | | |
| <27 kg/m2 | 80 (51.6%) | 260 (47.4%) | 119 (50.4%) | 221 (47.2%) | 198(50.3%) | 142 (45.8%) |
| >27 kg/m2 | 75 (48.4%) | 289 (52.6%) | 117 (49.6%) | 247 (52.8%) | 196(49.7%) | 168 (54.2%) |
| Age (Years) | | | | | | |
| <60 years | 81 (52.3%) | 304 (55.4%) | 120 (50.8%) | 265 (56.6%) | 186 (47.2%) | 199 (64.2%) |
| >60 years | 74 (47.7%) | 245 (44.6%) | 116 (49.2%) | 203 (43.4%) | 208 (52.8%) | 111 (35.8%) |
| Aortic Cross Clamp (AC) Time (minutes) | | | | | | |
| <37 minutes | 75 (48.4%) | 287 (52.3%) | 117 (49.6%) | 245 (52.4%) | 205 (52%) | 157 (50.6%) |
| >37 minutes | 80 (51.6%) | 262 (47.7%) | 119 (50.4%) | 223 (47.6%) | 189 (48%) | 153 (49.4%) |
| Cardiopulmonary Bypass (CBP) Time (minutes) | | | | | | |
| <58 minutes | 76 (49%) | 299 (54.5%) | 119 (50.4%) | 256 (54.7%) | 224 (56.9%) | 151 (48.7%) |
| >58 minutes | 79 (51%) | 250 (45.5%) | 117 (49.6%) | 212 (45.3%) | 170 (43.1%) | 159 (51.3%) |
| Left Ventricular Ejection Fraction (%age) | | | | | | |
| <55 % | 97 (62.6%) | 300 (54.6%) | 166 (70.3%) | 231 (49.4%) | 225 (57.1%) | 172 (55.5%) |
| >55% | 58 (37.4%) | 249 (45.4%) | 70 (29.7%) | 237 (50.6%) | 169 (42.9%) | 138 (44.5%) |
| Stay at Hospital (Days) | | | | | | |
| <7 Days | 97 (62.6%) | 367 (66.8%) | 143 (60.6%) | 321 (68.6%) | 242 (61.4%) | 222 (71.6%) |
| >7 Days | 58 (37.4%) | 182 (33.2%) | 93 (39.4%) | 147 (31.4%) | 152 (38.6%) | 88 (28.4%) |
| Prevalence of AKI was determined on basis of increase in Serum Creatinine values.  Prevalence across each variable is determined by performing a 2*2 contingency/ crosstabs in SPSS  AKI_Day2: acute kidney injury (AKI) prevalence measured by increase in values of serum creatinine on day two following surgery  AKI_Day7: acute kidney injury (AKI) prevalence measured by increase in values of serum creatinine on day seven following surgery  AKI_FollowUP_Day: acute kidney injury (AKI) prevalence measured by increase in values of serum creatinine on follow-up day  The threshold value for diagnosis of AKI was 0.3mg/dL increase in Serum Creatinine value when compared to baseline.  Day 2 : Post-surgical day two  Day 7 : Post-surgical day seven | | | | | | |
