# Supplementary material for: Assessment of renal function and prevalence of acute kidney injury following coronary artery bypass graft surgery and associated risk factors: A retrospective cohort study at a tertiary care hospital in Islamabad, Pakistan
Source: Medicine (Baltimore). 2023 Oct 20;102(42):e35482. doi: 10.1097/MD.0000000000035482 (PMC10589541; doi:10.1097/MD.0000000000035482)
Supplement: Supplementary file 6 [file medi-102-e35482-s006.docx]

Supplementary Table 6: Case processing summery for Kaplan Meier Curve (Log Rank)

| AKI stage | Total Number | Number of Events | Censored | |
| --- | --- | --- | --- | --- |
|  |  |  | N | %age |
| AKI stage measured by S.Cr value according to RIFLE criteria (Fractional_Rise_SCrDay2)* Days in Hospital. Corresponding Figure 6 | | | | |
| No Risk | 611 | 64 | 547 | 89.50% |
| Risk | 81 | 79 | 2 | 2.50% |
| Injury | 9 | 9 | 0 | 0.00% |
| Failure | 3 | 3 | 0 | 0.00% |
| Overall | 704 | 155 | 549 | 78.00% |
| AKI stage measured by S.Cr value according to RIFLE criteria (Fractional_Rise_SCrDay2)* Age in Years. Corresponding Figure 7 | | | | |
| No Risk | 611 | 64 | 547 | 89.50% |
| Risk | 81 | 79 | 2 | 2.50% |
| Injury | 9 | 9 | 0 | 0.00% |
| Failure | 3 | 3 | 0 | 0.00% |
| Overall | 704 | 155 | 549 | 78.00% |
|  | | | | |
| AKI stage according to RIFLE criteria (Fractional_Rise_SCrDay7) * Age in Years. Corresponding Figure 8 | | | | |
| No Risk | 571 | 106 | 465 | 81.40% |
| Risk | 116 | 113 | 3 | 2.60% |
| Injury | 13 | 13 | 0 | 0.00% |
| Failure | 4 | 4 | 0 | 0.00% |
| Overall | 704 | 236 | 468 | 66.50% |
|  | | | | |
| AKI stage according to RIFLE criteria (Fractional_Rise_SCrDay7)* Days in Hospital. Corresponding Figure 9 | | | | |
| No Risk | 571 | 106 | 465 | 81.40% |
| Risk | 116 | 113 | 3 | 2.60% |
| Injury | 13 | 13 | 0 | 0.00% |
| Failure | 4 | 4 | 0 | 0.00% |
| Overall | 704 | 236 | 468 | 66.50% |
|  | | | | |
| AKI stage according to RIFLE criteria (Fractional_Rise_SCrFUDay) * Follow-up in weeks. Corresponding Figure 10 | | | | |
| No Risk | 404 | 94 | 310 | 76.70% |
| Risk | 183 | 183 | 0 | 0.00% |
| Injury | 104 | 104 | 0 | 0.00% |
| Failure | 13 | 13 | 0 | 0.00% |
| Overall | 704 | 394 | 310 | 44.00% |
|  | | | | |
| AKI stage according to RIFLE criteria (Fractional_Rise_SCrFUDay) * Age in Years. Corresponding Figure 11 | | | | |
| No Risk | 404 | 94 | 310 | 76.70% |
| Risk | 183 | 183 | 0 | 0.00% |
| Injury | 104 | 104 | 0 | 0.00% |
| Failure | 13 | 13 | 0 | 0.00% |
| Overall | 704 | 394 | 310 | 44.00% |
| Fractional_Rise_SCrDay2: Acute rise in Value of Serum Creatinine Determined on post-surgical day two  Fractional_Rise_SCrDay7: Rise in Value of Serum Creatinine Determined on post-surgical day Seven  Fractional_Rise_SCrFUDay: Rise in Value of Serum Creatinine Determined on Follow-up Day | | | | |
