# Supplementary material for: Assessment of renal function and prevalence of acute kidney injury following coronary artery bypass graft surgery and associated risk factors: A retrospective cohort study at a tertiary care hospital in Islamabad, Pakistan
Source: Medicine (Baltimore). 2023 Oct 20;102(42):e35482. doi: 10.1097/MD.0000000000035482 (PMC10589541; doi:10.1097/MD.0000000000035482)
Supplement: Supplementary file 7 [file medi-102-e35482-s007.docx]

Supplementary Table 7: Log Rank (Mental-cox)

|  | Chi-Square | df | Sig. |
| --- | --- | --- | --- |
| Test of equality of survival distributions for the different levels of AKI stage measured by S.Cr value according to RIFLE criteria. AKI stage according to RIFLE criteria (Fractional_Rise_SCrDay2) * DIH | | | |
| Log Rank (Mantel-Cox) | 241.497 | 3 | .000 |
| Test of equality of survival distributions for the different levels of AKI stage according to RIFLE criteria (Fractional_Rise_SCrDay2). AKI stage according to RIFLE criteria (Fractional_Rise_SCrDay2) * Age | | | |
| Log Rank (Mantel-Cox) | 277.879 | 3 | .000 |
| Test of equality of survival distributions for the different levels of AKI stage according to RIFLE criteria (Fractional_Rise_SCrDay7) * Age | | | |
| Log Rank (Mantel-Cox) | 208.548 | 3 | .000 |
| Test of equality of survival distributions for the different levels of AKI stage according to RIFLE criteria (Fractional_Rise_SCrDay7) * DIH | | | |
| Log Rank (Mantel-Cox) | 154.081 | 3 | .000 |
| Test of equality of survival distributions for the different levels of AKI stage according to RIFLE criteria (Fractional Rise in S.Cr on follow-up day * Follow-up in weeks | | | |
| Log Rank (Mantel-Cox) | 211.336 | 3 | .000 |
| Test of equality of survival distributions for the different levels of AKI stage according to RIFLE criteria (Fractional Rise in S.Cr on follow-up day * Age | | | |
| Log Rank (Mantel-Cox) | 129.458 | 3 | .000 |
