# Supplementary material for: Assessment of renal function and prevalence of acute kidney injury following coronary artery bypass graft surgery and associated risk factors: A retrospective cohort study at a tertiary care hospital in Islamabad, Pakistan
Source: Medicine (Baltimore). 2023 Oct 20;102(42):e35482. doi: 10.1097/MD.0000000000035482 (PMC10589541; doi:10.1097/MD.0000000000035482)
Supplement: Supplementary file 8 [file medi-102-e35482-s008.docx]

Supplementary Table 8: Minimum and maximum value of variable in dataset, mean and standard deviation and median values of all the continuous variables

| Variable | Minimum | Maximum | Median | Mean | SD |
| --- | --- | --- | --- | --- | --- |
| Age | 32 | 79 | 60 | 59.74 | 7.94 |
| Days Stayed at Hospital | 1 | 30 | 7 | 7.54 | 3.091 |
| Body Mass Index | 18.6 | 39.9 | 27 | 27.52 | 3.7965 |
| Ejection Fraction | 27 | 93 | 55 | 54.3 | 13.038 |
| Aortic cross clamp time | 13 | 86 | 37 | 38.19 | 9.508 |
| Cardiopulmonary bypass time | 25 | 127 | 58 | 57.77 | 14.620 |
| S.Cr on day of surgery | 0.4 | 1.9 | 1.1 | 1.064 | 0.2504 |
| eGFR on day of surgery | 28 | 115 | 73 | 73.08 | 14.310 |
| B.Urea on day of surgery | 11 | 71 | 33 | 33.64 | 9.978 |
| S.Cr on day 2 | 0.4 | 2.0 | 1.2 | 1.255 | 0.2673 |
| eGFR on day 2 | 32 | 128 | 62 | 63.36 | 16.048 |
| B.Urea on day 2 | 14 | 85 | 32 | 34.59 | 10.145 |
| S.Cr on day 7 | 0.5 | 2.5 | 1.3 | 1.314 | 0.3320 |
| eGFR on day 7 | 27 | 121 | 57 | 60.49 | 17.772 |
| B.Urea on day 7 | 14 | 70 | 38 | 38.64 | 10.377 |
| S.Cr on follow-up day | 0.6 | 2.6 | 1.5 | 1.496 | 0.4391 |
| eGFR on follow-up day | 23 | 114 | 48 | 54.70 | 21.788 |
| S.Cr increase on day 2 | -0.60 | 1.0 | 0.2 | 0.1911 | 0.23451 |
| S.Cr increase on day 7 | -0.60 | 1.20 | 0.2 | 0.2500 | 0.26786 |
| S.Cr increase on follow-up day | -1.00 | 1.90 | 0.400 | 0.4327 | 0.45987 |
| Fractional S.Cr increase on day 2 | 0.44 | 3.50 | 1.1818 | 1.2171 | 0.30209 |
| Fractional S.Cr increase on day 7 | 0.56 | 3.75 | 1.2500 | 1.2670 | 0.32542 |
| Fractional S.Cr increases on follow-up day | 0.47 | 4.25 | 1.4000 | 1.4743 | 0.54676 |
| Percentage decrease in eGFR values on day 2 | -71.154 | 48.387 | 14.6250 | 12.22717 | 19.603398 |
| Percentage decrease in eGFR values day 7 | -71.154 | 66.667 | 18.82353 | 16.63087 | 21.000185 |
| Percentage decrease in eGFR values on follow-up day | -133.333 | 75.532 | 32.00000 | 23.24446 | 32.197128 |
| *Descriptive Statistics were measured using SPSSs  S.Cr: Serum Creatinine  eGFR: Estimated Glomerular Filtration Rate  Day 2 : Post-surgical day two  Day 7 : Post-surgical day seven | | | | | |
