# Supplementary material for: Assessment of renal function and prevalence of acute kidney injury following coronary artery bypass graft surgery and associated risk factors: A retrospective cohort study at a tertiary care hospital in Islamabad, Pakistan
Source: Medicine (Baltimore). 2023 Oct 20;102(42):e35482. doi: 10.1097/MD.0000000000035482 (PMC10589541; doi:10.1097/MD.0000000000035482)
Supplement: Supplementary file 9 [file medi-102-e35482-s009.docx]

Supplementary Table 9: Association between post-surgery Renal Function loss / renal damage over time and demographic, pre-clinical and clinical characteristics

|  | Renal Function_1 | | | | Renal Function_2 | | | |
| --- | --- | --- | --- | --- | --- | --- | --- | --- |
| Gender | Male | Female | Total | p-value | Male | Female | Total | p-value |
| Normal | 88 | 19 | 107 | 0.351 | 41 | 9 | 50 | 0.788 |
| Mild loss | 411 | 66 | 477 |  | 291 | 55 | 346 |  |
| Mild-moderate loss | 83 | 21 | 104 |  | 183 | 35 | 218 |  |
| Moderate-severe loss | 10 | 3 | 13 |  | 79 | 11 | 90 |  |
| Severe loss | 2 | 1 | 3 |  |  |  |  |  |
| **Total** | **594** | **110** | **704** |  | **594** | **110** | **704** |  |
| Age (Years) | < 60 | > 60 |  |  | < 60 | > 60 | Total |  |
| Normal | 65 | 42 | 107 | 0.030 | 39 | 11 | 50 | 0.000 |
| Mild loss | 266 | 211 | 477 |  | 204 | 142 | 346 |  |
| Mild-moderate loss | 44 | 60 | 104 |  | 100 | 118 | 218 |  |
| Moderate-severe loss | 7 | 6 | 13 |  | 42 | 48 | 90 |  |
| Severe loss | 3 | 0 | 3 |  |  |  |  |  |
| **Total** | **385** | **319** | **704** |  | **385** | **319** | **704** |  |
| Diagnosis | TVCAD | TVCAD+ LMS |  |  | TVCAD | TVCAD+ LMS | Total |  |
| Normal | 63 | 44 | 107 | 0.451 | 28 | 22 | 50 | 0.190 |
| Mild loss | 326 | 151 | 477 |  | 241 | 105 | 346 |  |
| Mild-moderate loss | 71 | 33 | 104 |  | 146 | 72 | 218 |  |
| Moderate-severe loss | 9 | 4 | 13 |  | 56 | 34 | 90 |  |
| Severe loss | 2 | 1 | 3 |  |  |  |  |  |
| **Total** | **471** | **233** | **704** |  | **471** | **233** | **704** |  |
| DIH | < 7 | > 7 |  |  | < 7 | > 7 | Total |  |
| Normal | 69 | 38 | 107 | 0.953 | 37 | 13 | 50 | 0.295 |
| Mild loss | 319 | 158 | 477 |  | 233 | 113 | 346 |  |
| Mild-moderate loss | 66 | 38 | 104 |  | 134 | 84 | 218 |  |
| Moderate-severe loss | 8 | 5 | 13 |  | 60 | 30 | 90 |  |
| Severe loss | 2 | 1 | 3 |  |  |  |  |  |
| **Total** | **464** | **240** | **704** |  | **464** | **240** | **704** |  |
| BMI Kg/m2 | < 27 | > 27 |  |  | < 27 | > 27 | Total |  |
| Normal | 54 | 53 | 107 | 0.823 | 15 | 35 | 50 | 0.028 |
| Mild loss | 224 | 253 | 477 |  | 175 | 171 | 346 |  |
| Mild-moderate loss | 54 | 50 | 104 |  | 101 | 117 | 218 |  |
| Moderate-severe loss | 7 | 6 | 13 |  | 49 | 41 | 90 |  |
| Severe loss | 1 | 2 | 3 |  |  |  |  |  |
| **Total** | **340** | **364** | **704** |  | **340** | **364** | **704** |  |
| LVEF in %age | < 55 | > 55 |  |  | < 55 | > 55 | Total |  |
| Normal | 41 | 66 | 107 | 0.000 | 17 | 33 | 50 | 0.000 |
| Mild loss | 268 | 209 | 477 |  | 160 | 186 | 346 |  |
| Mild-moderate loss | 75 | 29 | 104 |  | 142 | 76 | 218 |  |
| Moderate-severe loss | 10 | 3 | 13 |  | 78 | 12 | 90 |  |
| Severe loss | 3 | 0 | 3 |  |  |  |  |  |
| **Total** | **397** | **307** | **704** |  | **397** | **307** | **704** |  |
| Hypertension | Present | Absent | Total |  | Present | Absent | Total |  |
| Normal | 77 | 30 | 107 | 0.095 | 34 | 16 | 50 | 0.767 |
| Mild loss | 309 | 168 | 477 |  | 228 | 118 | 346 |  |
| Mild-moderate loss | 81 | 23 | 104 |  | 152 | 66 | 218 |  |
| Moderate-severe loss | 8 | 5 | 13 |  | 63 | 27 | 90 |  |
| Severe loss | 2 | 1 | 3 |  |  |  |  |  |
| **Total** | **477** | **227** | **704** |  | **477** | **227** | **704** |  |
| Diabetes Mellitus | Present | Absent | Total |  | Present | Absent | Total |  |
| Normal | 61 | 46 | 107 | 0.626 | 32 | 18 | 50 | 0.358 |
| Mild loss | 274 | 203 | 477 |  | 204 | 142 | 346 |  |
| Mild-moderate loss | 64 | 40 | 104 |  | 118 | 100 | 218 |  |
| Moderate-severe loss | 10 | 3 | 13 |  | 57 | 33 | 90 |  |
| Severe loss | 2 | 1 | 3 |  |  |  |  |  |
| **Total** | **411** | **293** | **704** |  | **411** | **293** | **704** |  |
| IHD | Present | Absent | Total |  | Present | Absent | Total |  |
| Normal | 81 | 26 | 107 | 0.001 | 32 | 18 | 50 | 0.147 |
| Mild loss | 336 | 141 | 477 |  | 255 | 91 | 346 |  |
| Mild-moderate loss | 80 | 24 | 104 |  | 159 | 59 | 218 |  |
| Moderate-severe loss | 3 | 10 | 13 |  | 57 | 33 | 90 |  |
| Severe loss | 3 | 0 | 3 |  |  |  |  |  |
| Total | 503 | 201 | 704 |  | 503 | 201 | 704 |  |
| CKD | Present | Absent | Total |  | Present | Absent | Total |  |
| Normal | 4 | 103 | 107 | 0.034 | 0 | 50 | 50 | 0.001 |
| Mild loss | 24 | 453 | 477 |  | 17 | 329 | 346 |  |
| Mild-moderate loss | 10 | 94 | 104 |  | 11 | 207 | 218 |  |
| Moderate-severe loss | 2 | 11 | 13 |  | 13 | 77 | 90 |  |
| Severe loss | 1 | 2 | 3 |  |  |  |  |  |
| **Total** | **41** | **663** | **704** |  | **41** | **663** | **704** |  |
| Asthma | Present | Absent | Total |  | Present | Absent | Total |  |
| Normal | 2 | 105 | 107 | 0.654 | 1 | 49 | 50 | 0.427 |
| Mild loss | 5 | 472 | 477 |  | 4 | 342 | 346 |  |
| Mild-moderate loss | 3 | 101 | 104 |  | 5 | 213 | 218 |  |
| Moderate-severe loss | 0 | 13 | 13 |  | 0 | 90 | 90 |  |
| Severe loss | 0 | 3 | 3 |  |  |  |  |  |
| **Total** | **10** | **694** | **704** |  | **10** | **694** | **704** |  |
| CVA | Present | Absent | Total |  | Present | Absent | Total |  |
| Normal | 2 | 105 | 107 | 0.769 | 0 | 50 | 50 | 0.117 |
| Mild loss | 14 | 463 | 477 |  | 7 | 339 | 346 |  |
| Mild-moderate loss | 4 | 100 | 104 |  | 11 | 207 | 218 |  |
| Moderate-severe loss | 1 | 12 | 13 |  | 3 | 87 | 90 |  |
| Severe loss | 0 | 3 | 3 |  |  |  |  |  |
| **Total** | **21** | **683** | **704** |  | **21** | **683** | **704** |  |
| ACC_Time (minutes) | < 37 | > 37 | Total |  | < 37 | > 37 | Total |  |
| Normal | 57 | 50 | 107 | 0.819 | 23 | 27 | 50 | 0.626 |
| Mild loss | 246 | 231 | 477 |  | 182 | 164 | 346 |  |
| Mild-moderate loss | 50 | 54 | 104 |  | 115 | 103 | 218 |  |
| Moderate-severe loss | 8 | 5 | 13 |  | 42 | 48 | 90 |  |
| Severe loss | 1 | 2 | 3 |  |  |  |  |  |
| **Total** | **362** | **342** | **704** |  | **362** | **342** | **704** |  |
| CPB_Time (minutes) | < 58 | > 58 | Total |  | < 58 | > 58 | Total |  |
| Normal | 52 | 55 | 107 | 0.393 | 23 | 27 | 50 | 0.613 |
| Mild loss | 255 | 222 | 477 |  | 187 | 159 | 346 |  |
| Mild-moderate loss | 56 | 48 | 104 |  | 120 | 98 | 218 |  |
| Moderate-severe loss | 10 | 3 | 13 |  | 45 | 45 | 90 |  |
| Severe loss | 2 | 1 | 3 |  | - | - | - |  |
| **Total** | **375** | **329** | **704** |  | **375** | **329** | **704** |  |
| Smoking | Smoker | Non-smoker | Total |  | Smoker | Non-smoker | Total |  |
| Normal | 48 | 59 | 107 | 0.577 | 19 | 31 | 50 | 0.545 |
| Mild loss | 213 | 264 | 477 |  | 165 | 181 | 346 |  |
| Mild-moderate loss | 53 | 51 | 104 |  | 96 | 122 | 218 |  |
| Moderate-severe loss | 8 | 5 | 13 |  | 43 | 47 | 90 |  |
| Severe loss | 1 | 2 | 3 |  | - | - | - |  |
| **Total** | **323** | **381** | **704** |  | **323** | **381** | **704** |  |
|  | **Renal Function_3** | | | | **Renal Function_4** | | | |
| Gender | Male | Female | Total | p-value | Male | Female | Total | p-value |
| Normal | 30 | 8 | 38 | 0.030 | 55 | 4 | 59 | 0.013 |
| Mild loss | 243 | 58 | 301 |  | 152 | 41 | 193 |  |
| Mild-moderate loss | 202 | 24 | 226 |  | 131 | 28 | 159 |  |
| Moderate-severe loss | 109 | 16 | 125 |  | 219 | 28 | 247 |  |
| Severe loss | 10 | 4 | 14 |  | 37 | 9 | 46 |  |
| **Total** | 594 | 110 | 704 |  | 594 | 110 | 704 |  |
| Age (Years) | < 60 | > 60 |  |  | < 60 | > 60 | Total |  |
| Normal | 24 | 14 | 38 | 0.013 | 46 | 13 | 59 | 0.000 |
| Mild loss | 181 | 120 | 301 |  | 125 | 68 | 193 |  |
| Mild-moderate loss | 119 | 107 | 226 |  | 96 | 63 | 159 |  |
| Moderate-severe loss | 53 | 72 | 125 |  | 106 | 141 | 247 |  |
| Severe loss | 8 | 6 | 14 |  | 12 | 34 | 46 |  |
| **Total** | 385 | 319 | 704 |  | 385 | 319 | 704 |  |
| Diagnosis | TVCAD | TVCAD+ LMS |  |  | TVCAD | TVCAD+ LMS | Total |  |
| Normal | 23 | 15 | 38 | 0.183 | 40 | 19 | 59 | 0.113 |
| Mild loss | 210 | 91 | 301 |  | 123 | 70 | 193 |  |
| Mild-moderate loss | 156 | 70 | 226 |  | 114 | 45 | 159 |  |
| Moderate-severe loss | 74 | 51 | 125 |  | 170 | 77 | 247 |  |
| Severe loss | 8 | 6 | 14 |  | 24 | 22 | 46 |  |
| **Total** | 471 | 233 | 704 |  | 471 | 233 | 704 |  |
| DIH | < 7 | > 7 |  |  | < 7 | > 7 | Total |  |
| Normal | 29 | 9 | 38 | 0.337 | 45 | 14 | 59 | 0.073 |
| Mild loss | 206 | 95 | 301 |  | 136 | 57 | 193 |  |
| Mild-moderate loss | 143 | 83 | 226 |  | 105 | 54 | 159 |  |
| Moderate-severe loss | 78 | 47 | 125 |  | 148 | 99 | 247 |  |
| Severe loss | 8 | 6 | 14 |  | 30 | 16 | 46 |  |
| **Total** | 464 | 240 | 704 |  | 464 | 240 | 704 |  |
| BMI Kg/m2 | < 27 | > 27 |  |  | < 27 | > 27 | Total |  |
| Normal | 13 | 25 | 38 | 0.214 | 24 | 35 | 59 | 0.682 |
| Mild loss | 144 | 157 | 301 |  | 91 | 102 | 193 |  |
| Mild-moderate loss | 110 | 116 | 226 |  | 76 | 83 | 159 |  |
| Moderate-severe loss | 68 | 57 | 125 |  | 125 | 122 | 247 |  |
| Severe loss | 5 | 9 | 14 |  | 24 | 22 | 46 |  |
| **Total** | 340 | 364 | 704 |  | 340 | 364 | 704 |  |
| LVEF in %age | < 55 | > 55 |  |  | < 55 | > 55 | Total |  |
| Normal | 16 | 22 | 38 | 0.000 | 30 | 29 | 59 | 0.817 |
| Mild loss | 93 | 208 | 301 |  | 105 | 88 | 193 |  |
| Mild-moderate loss | 168 | 58 | 226 |  | 93 | 66 | 159 |  |
| Moderate-severe loss | 107 | 18 | 125 |  | 142 | 105 | 247 |  |
| Severe loss | 13 | 1 | 14 |  | 27 | 19 | 46 |  |
| **Total** | 397 | 307 | 704 |  | 397 | 307 | 704 |  |
| Hypertension | Present | Absent | Total |  | Present | Absent | Total |  |
| Normal | 26 | 12 | 38 | 0.789 | 40 | 19 | 59 | 0.523 |
| Mild loss | 198 | 103 | 301 |  | 129 | 64 | 193 |  |
| Mild-moderate loss | 154 | 72 | 226 |  | 100 | 59 | 159 |  |
| Moderate-severe loss | 88 | 37 | 125 |  | 175 | 72 | 247 |  |
| Severe loss | 11 | 3 | 14 |  | 33 | 13 | 46 |  |
| **Total** | 477 | 227 | 704 |  | 477 | 227 | 704 |  |
| Diabetes Mellitus | Present | Absent | Total |  | Present | Absent | Total |  |
| Normal | 26 | 12 | 38 | 0.011 | 39 | 20 | 59 | 0.043 |
| Mild loss | 183 | 118 | 301 |  | 118 | 75 | 193 |  |
| Mild-moderate loss | 112 | 114 | 226 |  | 80 | 79 | 159 |  |
| Moderate-severe loss | 79 | 46 | 125 |  | 141 | 106 | 247 |  |
| Severe loss | 11 | 3 | 14 |  | 33 | 13 | 46 |  |
| **Total** | 411 | 293 | 704 |  | 411 | 293 | 704 |  |
| IHD | Present | Absent | Total |  | Present | Absent | Total |  |
| Normal | 21 | 17 | 38 | 0.137 | 43 | 16 | 59 | 0.489 |
| Mild loss | 218 | 83 | 301 |  | 138 | 55 | 193 |  |
| Mild-moderate loss | 169 | 57 | 226 |  | 109 | 50 | 159 |  |
| Moderate-severe loss | 85 | 40 | 125 |  | 184 | 63 | 247 |  |
| Severe loss | 10 | 4 | 14 |  | 29 | 17 | 46 |  |
| Total | 503 | 201 | 704 |  | 503 | 201 | 704 |  |
| CKD | Present | Absent | Total |  | Present | Absent | Total |  |
| Normal | 2 | 36 | 38 | 0.102 | 2 | 57 | 59 | 0.026 |
| Mild loss | 11 | 290 | 301 |  | 11 | 182 | 193 |  |
| Mild-moderate loss | 14 | 212 | 226 |  | 12 | 147 | 159 |  |
| Moderate-severe loss | 12 | 113 | 125 |  | 9 | 238 | 247 |  |
| Severe loss | 2 | 12 | 14 |  | 7 | 39 | 46 |  |
| **Total** | 41 | 663 | 704 |  | 41 | 663 | 704 |  |
| Asthma | Present | Absent | Total |  | Present | Absent | Total |  |
| Normal | 1 | 37 | 38 | 0.673 | 0 | 59 | 59 | 0.043 |
| Mild loss | 3 | 298 | 301 |  | 2 | 191 | 193 |  |
| Mild-moderate loss | 5 | 221 | 226 |  | 0 | 159 | 159 |  |
| Moderate-severe loss | 1 | 124 | 125 |  | 8 | 239 | 247 |  |
| Severe loss | 0 | 14 | 14 |  | 0 | 46 | 46 |  |
| **Total** | 10 | 694 | 704 |  | 10 | 694 | 704 |  |
| CVA | Present | Absent | Total |  | Present | Absent | Total |  |
| Normal | 0 | 38 | 38 | 0.184 | 1 | 58 | 59 | 0.920 |
| Mild loss | 12 | 289 | 301 |  | 5 | 188 | 193 |  |
| Mild-moderate loss | 3 | 223 | 226 |  | 5 | 154 | 159 |  |
| Moderate-severe loss | 6 | 119 | 125 |  | 9 | 238 | 247 |  |
| Severe loss | 0 | 14 | 14 |  | 1 | 45 | 46 |  |
| **Total** | 21 | 683 | 704 |  | 21 | 683 | 704 |  |
| ACC_Time (minutes) | < 37 | > 37 | Total |  | < 37 | > 37 | Total |  |
| Normal | 20 | 18 | 38 | 0.825 | 28 | 31 | 59 | 0.026 |
| Mild loss | 154 | 147 | 301 |  | 110 | 83 | 193 |  |
| Mild-moderate loss | 121 | 105 | 226 |  | 68 | 91 | 159 |  |
| Moderate-severe loss | 59 | 66 | 125 |  | 137 | 110 | 247 |  |
| Severe loss | 8 | 6 | 14 |  | 19 | 27 | 46 |  |
| **Total** | 362 | 342 | 704 |  | 362 | 342 | 704 |  |
| CPB_Time (minutes) | < 58 | > 58 | Total |  | < 58 | > 58 | Total |  |
| Normal | 17 | 21 | 38 | 0.842 | 20 | 39 | 59 | 0.026 |
| Mild loss | 164 | 137 | 301 |  | 110 | 83 | 193 |  |
| Mild-moderate loss | 119 | 107 | 226 |  | 82 | 77 | 159 |  |
| Moderate-severe loss | 67 | 58 | 125 |  | 136 | 111 | 247 |  |
| Severe loss | 8 | 6 | 14 |  | 27 | 19 | 46 |  |
| **Total** | 375 | 329 | 704 |  | 375 | 329 | 704 |  |
| Smoking | Smoker | Non-smoker | Total |  | Smoker | Non-smoker | Total |  |
| Normal | 19 | 19 | 38 | 0.654 | 32 | 27 | 59 | 0.342 |
| Mild loss | 136 | 165 | 301 |  | 87 | 106 | 193 |  |
| Mild-moderate loss | 103 | 123 | 226 |  | 66 | 93 | 159 |  |
| Moderate-severe loss | 61 | 64 | 125 |  | 120 | 127 | 247 |  |
| Severe loss | 4 | 10 | 14 |  | 18 | 28 | 46 |  |
| **Total** | **323** | **381** | **704** |  | **323** | **381** | **704** |  |
| **Renal Function_1:** Stage of loss of Renal Function at the Day of Surgery (Most recent values prior to surgery)  **Renal Function_2:** Stage of loss of Renal Function at Post-surgical Day two  **Renal Function_3:** Stage of loss of Renal Function at Post-surgical Day seven  **Renal Function_4:** Stage of loss of Renal Function at Follow-up Visit  *The stage of loss of Renal Function is determined by the values of eGFR obtained at the day of surgery, Second day following surgery, Seventh day following surgery and at the Follow-up visit.  HTN: hypertension, DM: diabetes mellitus, IHD: ischemic heart disease, CKD: chronic kidney disease, CVA: cerebr-o-vascular accident, BMI: body mass index, EF: ejection fraction, ACC_T: aortic cross clamp time and CPB: cardiopulmonary bypass time | | | | | | | | |
