# Supplementary material for: Assessment of renal function and prevalence of acute kidney injury following coronary artery bypass graft surgery and associated risk factors: A retrospective cohort study at a tertiary care hospital in Islamabad, Pakistan
Source: Medicine (Baltimore). 2023 Oct 20;102(42):e35482. doi: 10.1097/MD.0000000000035482 (PMC10589541; doi:10.1097/MD.0000000000035482)
Supplement: Supplementary file 10 [file medi-102-e35482-s010.docx]

Supplementary Table 10: CKD and Renal Damage

| Chronic Kidney Disease |  | No loss | | Mild loss | | Mild-moderate | | Moderate-severe | | Severe | |  | | p-value |
| --- | --- | --- | --- | --- | --- | --- | --- | --- | --- | --- | --- | --- | --- | --- |
|  | present | 4 | | 24 | | 10 | | 2 | | 1 | | 41 | |  |
|  | absent | 103 | | 453 | | 94 | | 11 | | 2 | | 663 | |  |
| Total | | 107 | | 477 | | 104 | | 13 | | 3 | | 704 | | 0.034 |
|  | present | 0 | | 17 | | 11 | | 13 | | 0 | | 41 | |  |
|  | absent | 50 | | 329 | | 207 | | 77 | | 0 | | 663 | |  |
| Total | | 50 | | 346 | | 218 | | 90 | | 0 | | 704 | | 0.001 |
|  | present | 2 | | 11 | | 14 | | 12 | | 2 | | 41 | |  |
|  | absent | 36 | | 290 | | 212 | | 113 | | 12 | | 663 | |  |
| Total | | 38 | | 301 | | 226 | | 125 | | 14 | | 704 | | 0.102 |
|  | present | 2 | | 11 | | 12 | | 9 | | 7 | | 41 | |  |
|  | absent | 57 | | 182 | | 147 | | 238 | | 39 | | 663 | |  |
| Total | | 59 | | 193 | | 159 | | 247 | | 46 | | 704 | | 0.026 |
|  | | | Sig. (2-tailed) | | present | | absent | | Mean Difference | | 95% Confidence Interval of the Difference | | | |
|  |  |  |  |  |  |  |  |  |  |  | Lower | | Upper | |
| S_Cr pre-operative values | | | .001 | | 1.1927 | | 1.0558 | | .13692 | | .05841 | | .21544 | |
| S_Cr values at Postsurgical Day 2 | | | .001 | | 1.3902 | | 1.2465 | | .14379 | | .05994 | | .22764 | |
| Postsurgical Day 7 S_Cr values | | | .001 | | 1.4829 | | 1.3033 | | .17961 | | .07548 | | .28373 | |
| S_Cr values at Follow Up Day | | | .813 | | 1.5122 | | 1.4955 | | .01672 | | -.12212 | | .15556 | |
